# Spillover modes in multiplex games: double-edged effects on cooperation and their coevolution

**DOI:** 10.1038/s41598-018-25025-3

**Published:** 2018-05-02

**Authors:** Tommy Khoo, Feng Fu, Scott Pauls

**Affiliations:** 1 Department of Mathematics, Dartmouth College, Hanover, NH 03755 USA; 2 Department of Biomedical Data Science, Geisel School of Medicine, Dartmouth College, Hanover, NH 03755 USA

## Abstract

In recent years, there has been growing interest in studying games on multiplex networks that account for interactions across linked social contexts. However, little is known about how potential cross-context interference, or spillover, of individual behavioural strategy impact overall cooperation. We consider three plausible spillover modes, quantifying and comparing their effects on the evolution of cooperation. In our model, social interactions take place on two network layers: repeated interactions with close neighbours in a lattice, and one-shot interactions with random individuals. Spillover can occur during the learning process with accidental cross-layer strategy transfer, or during social interactions with errors in implementation. Our analytical results, using extended pair approximation, are in good agreement with extensive simulations. We find double-edged effects of spillover: increasing the intensity of spillover can promote cooperation provided cooperation is favoured in one layer, but too much spillover is detrimental. We also discover a bistability phenomenon: spillover hinders or promotes cooperation depending on initial frequencies of cooperation in each layer. Furthermore, comparing strategy combinations emerging in each spillover mode provides good indication of their co-evolutionary dynamics with cooperation. Our results make testable predictions that inspire future research, and sheds light on human cooperation across social domains.

## Introduction

The ubiquity of cooperation in human societies and nature is a puzzling phenomenon^1–4^. At first glance, cooperation seems unlikely: cooperators incur cost in providing benefits to others, while opportunistic individuals can reap rewards without returning the favour^5^. Nonetheless, cooperation can arise in structured populations through the mechanism of network reciprocity^6–8^. This basic observation drove deeper investigations into reciprocity in structured populations^9–12^.

Real world networks are often interdependent, where a small perturbation to one network can trigger a chain of events that results in cataclysmic effects on both networks^13^. Taking the importance of such interconnectedness into account has led to a recent boom in the study of multiplex networks^14,15^. In the same vein, evolutionary games on multiplex networks are attracting increasing attention (we refer readers to^16^ for a review).

Various mechanisms have been proposed to associate different evolutionary games taking place on otherwise disjoint networks. One utility function approach incorporates payoffs accumulated across games on different networks into each strategic decision^17,18^. An alternative approach allows strategic behaviour to be transmitted from one setting to another through peer influence and social learning^19,20^.

In this paper, we draw inspiration from empirical results^21–25^, which suggest that norms and heuristics cultivated during repeated interactions could “spill over” to affect decision making in one-shot situations.

In two experiments^21,24^, when subjects first participate in the iterated prisoner’s dilemma (IPD)^26^, they observed greater cooperation and prosocial behaviour in subsequent one-shot games. A similar increase in prosocial behaviour follows a repeated public goods game with conditions favourable for cooperation^25^. Relatedly, cooperation levels rose when switching from an IPD with a large continuation probability to one with a small continuation probability^22^, as well as when switching from playing an IPD with a fixed partner to playing an IPD in which every iteration was played with a random partner^23^.

The hypothesized explanation^21,27,28^ for these phenomena is that repeated interactions foster cooperative heuristics or norms in participants which then affected subsequent one-shot games^4,29^. Although recent endeavours^27,30,31^ studied spillover using an evolutionary framework, systematic exploration of spillover mechanisms through the lens of games on multiplex networks are still lacking, with the exception of a recent work^32^.

**Figure 1:**
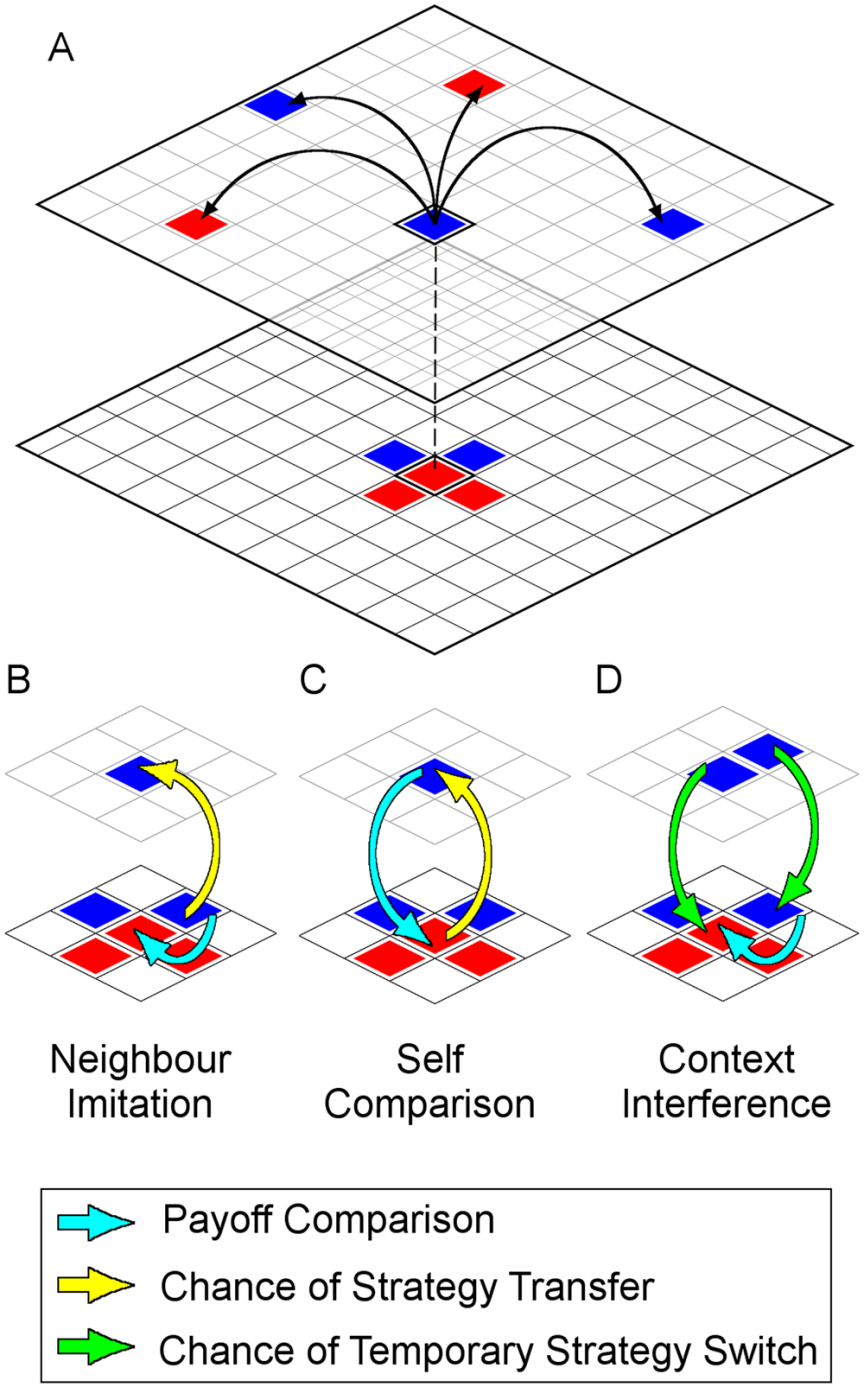
Spillover modes in multiplex games. In (**A**) On the bottom layer, individuals play the iterated prisoner’s dilemma on a square lattice with periodic boundary. On the top layer, they play the one shot prisoner’s dilemma on a random regular network with degree four. Individuals compare payoffs with neighbours within the layer, to decide whether to adopt a neighbour’s strategy in that layer. In addition, there is a probability of using one of the three spillover modes instead, to adopt strategies from another layer. For (**B**) neighbour imitation mode, the individual compares payoff with neighbours on the same layer, and applies adopted strategies to the opposite layer. In (**C**) self comparison mode, the individual compares her normalized payoffs on each layer, and decides whether to implement her strategy from one layer in another. For (**D**) context interference mode, the individual is susceptible to temporary interference from mistaking the context of the interaction, which results in temporarily using strategies from the opposite layer.

We fill this gap by modelling spillover as strategy interference between layers on a multiplex network (see Fig. 1A). Instead of the unstructured populations seen in the experiments, our *n* individuals participate in two layers of interactions with population structure that emulate recurring close proximity and distant one-shot contacts.

We represent recurring close proximity contacts using a square periodic lattice on the bottom layer where agents play *m* rounds of the IPD with their neighbours using two possible strategies *C* or *D*. Here, *C* refers to tit-for-tat (TFT), where individuals cooperates on the first iteration and play the opponent’s previous strategy for future iterations, and *D* stands for always defecting (ALLD) during every iteration. While other strategies are possible, we focus on these two classic strategies^33,34^ for simplicity. If two players play *C*, they both receive a payoff of *m*(*b* − *c*). If they both play *D*, they both receive 0. If one plays *C* and the other plays *D*, the *C* player gets a payoff of −*c* while the *D* player gets *b*.

On the top layer, the same agents, randomly connected to four different agents during every run, play the (one-shot) prisoner’s dilemma^35^ (PD) as a proxy for distant one-shot contacts. Here, agents choose to cooperate (*C*) or defect (*D*). Two cooperators will both receive a payoff of *b* − *c*, while two defectors both receives 0. If one cooperates and the other defects, the cooperator gets a payoff of −*c* while the defector gets *b*.

We propose three modes for spillover. In the first, which we call neighbour imitation spillover (NIS) mode Fig. 1B, individuals on one layer may imitate the strategic behaviour (C or D) of a neighbour on the opposite layer. In the second, self comparison spillover (SCS) mode Fig. 1C, individuals compare their payoffs between layers and learn from their experience. Finally, for the third context interference spillover (CIS) mode, illustrated in Fig. 1D, individuals may make a temporary mistake and apply their strategic behaviour from another layer. A parameter *p* determines the frequency of spillover occurrences in each case, and hence is a proxy for the strength of the spillover effect.

Although the strategy sets differ in the IPD and PD, we allow cooperative (C) and defective (D) behaviour to be transmitted through spillover in order to model experimental observations. Here, we do not account for the fine detail interactions between games^32^ but instead focus on the coarse grain interface of behavioural norms and social heuristics acquired through two different social contexts. Furthermore, we model repeated interactions with close proximity contacts as occurring with greater frequency than distant one-shot contacts, and hence we compare the *m* rounds IPD to the one round PD. Finally, we only use intra-layer payoffs in this part of our investigation in order to keep the two social contexts distinct, as well as to restrict cross context interference to occur only through our three spillover modes.

Through these three modes, we amalgamate key ideas from prior work. NIS and SCS encapsulates the notion of individuals making a mistake in learning a potentially suboptimal strategy from a different social setting^19,20^, either through their own experience or by interacting with others. CIS captures the idea of individuals making implementation mistakes due to confounding two different social settings^27,30,31^. We also note that our spillover mechanisms model mistakes occurring between distinct network games, as opposed to random errors modelled by mechanisms such as weak selection and mutation.

Our main finding is that cooperation depends subtly on the strength *p* and the initial level of cooperation on both the layers - a double-edged effect where different combinations encourage or discourage cooperation. These results expanded upon previous work containing an alternative formulation of NIS^20^, which used two one-shot games to investigate neighbour imitation and found that there is an intermediate optimal frequency for cooperation in one of the games, in the case of a well-mixed population. We also advance their results for unstructured populations by using pair approximation^36^ to derive analytical solutions that incorporate population structure, and demonstrate the effectiveness of our solutions with extensive simulations. These solutions allow us to study both the macroscopic overall cooperation level, as well as microscopic details regarding strategy combinations.

Finally, we analyse the three spillover modes as they coevolve with cooperation in the presence of mutation. Our findings suggest that transient implementation mistakes (CIS) outperforms mechanisms under which individuals might learn and retain suboptimal strategies (NIS, SCS). On the other hand, when we allow repeated local interactions to play a larger role during spillover conditions become more conducive to cooperation, making it less punishing to make learning mistakes with long lasting impact. This effect allows more deliberate mechanisms that promotes cooperation on both layers, like NIS and SCS, to thrive.

## Results

**Figure 2:**
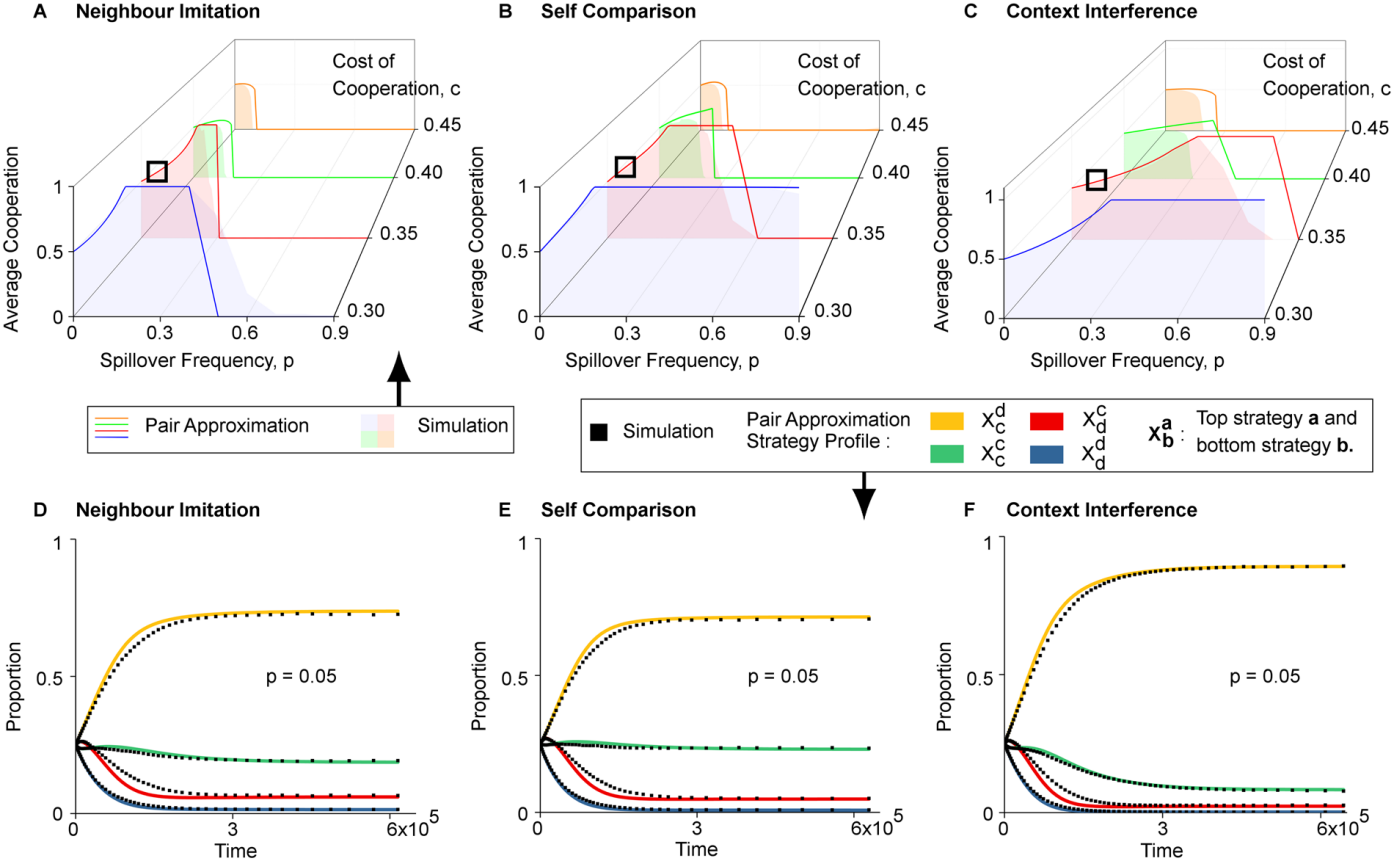
Double-edged effects of spillover. (**A**–**C**) Shows simulation and pair approximation results for average proportion of cooperators for each spillover mode (**A**) neighbour imitation, (**B**) self comparison and (**C**) context interference. As shown in more details by (**D**–**F**), analytical and simulation results are in good agreement at low frequencies of spillover *p*. For some parameter combinations, a double-edge effect of spillover on cooperation reveals itself in the form of an initial increase in cooperation with *p*, before a subsequent decline after an optimal *p*. (**D**–**F**) presents microscopic details of strategy profile proportions associated with each spillover mode, taken from (**A**–**C**) at parameter combinations $(\square )$. Self comparison has the largest proportion of individuals cooperating on both layers (green line), while context interference produces the largest proportion of individuals cooperating on the bottom layer and defecting on the top layer (yellow line). Parameters: *n* = 3600, *m* = 4, *β* = 0.2, *b* = 1, *α* = 0.5. Simulations: 6 × 10^6^ time steps, averaged over 100 runs. *p* has step size 0.01 from *p* = 0 to 0.4, and step size 0.1 otherwise.

We start our exploration by examining how the strength of the spillover effect impacts cooperation. To accomplish this, we produced simulation and pair approximation results for a range of parameter combinations. We present these in Fig. 2, with individual plots for each mode (NIS Fig. 2A, SCS Fig. 2B and CIS Fig. 2C).

The most striking feature of Fig. 2 is the existence of an optimal value of *p* which maximizes the average cooperation level in the multiplex network. Average cooperation initially increases with *p*, before reaching the optimal value and subsequently plummeting. This demonstrates a double-edged effect of spillover: a little spillover between the two social settings allows cooperators on the repeated local interaction layer to exert their influence on the distant one-shot contact layer and provides a boost to overall cooperation. On the other hand, a spillover effect that is too strong leads to too much influence by defectors on the distant one-shot contacts layer, and is deleterious to cooperation.

We note that conditions that are overly favourable or hostile to cooperation will lead to one layer overwhelming the other and consequently a rapid monotonic rise or decline in cooperation, instead of an intermediate optimal value of *p* (see Supplementary Fig. S1). Similarly, when both layers are analysed with the same game and parameters (both IPD or both PD), the overall conditions are too hostile for cooperation to thrive in the presence of spillover, and average cooperation in the multiplex decline with any increase in *p* (see Supplementary Figs S2 and S3).

Another feature of Fig. 2 is that CIS appears to be more resilient to this double-edge effect than NIS and SCS as cooperation levels for CIS tend to be higher than the other two. This is due to the fact that while implementation mistakes occur under CIS, payoff comparison always occurs on the layer on which the strategies are adopted. Hence, individuals have an easier time learning correct strategies, and implementation mistakes need not have a prolonged impact. On the other hand, under NIS and SCS, individuals directly learn and adopt strategies across layers, resulting in mistakes that have larger, long term repercussions on cooperation.

Next, we examine fine details for several parameter combinations from Fig. 2A–C highlighted with squares. These microscopic details (Fig. 2D–F) show the proportions of individuals for each possible strategy combination for the top and bottom layer. For simplicity, we let ${X}_{b}^{a}$ be the proportion of individuals playing strategy *a* on the top layer and *b* on the bottom layer.

Figure 2D–F demonstrates that for small values of *p*, there is excellent agreement between simulation and our pair approximation results at even the microscopic level (detailed equations are presented in the SI). The most outstanding feature is that context interference spillover mode has much higher ${X}_{c}^{d}$ than the other modes, as seen in Fig. 2F. This happens because, the parameter combinations in Fig. 2 leads to almost all *C* on the bottom layer and almost all *D* on the top layer. As we saw above, in CIS, players learn strategies more easily compared to NIS and SCS, so individuals can learn the optimal strategy of playing *D* on the bottom layer and playing *C* on the top layer.

On the other hand, SCS (Fig. 2E) stands out as having the highest proportion of individuals who are cooperators on both layers, with NIS (Fig. 2D) coming in a close second. In both cases, individuals are adopting spillover strategies that their neighbours or themselves have been successfully using within the opposite layer. This leads to a higher level of cooperation in the one-shot PD layer due to individuals learning and retaining the suboptimal strategy of playing *C* on that layer.

**Figure 3:**
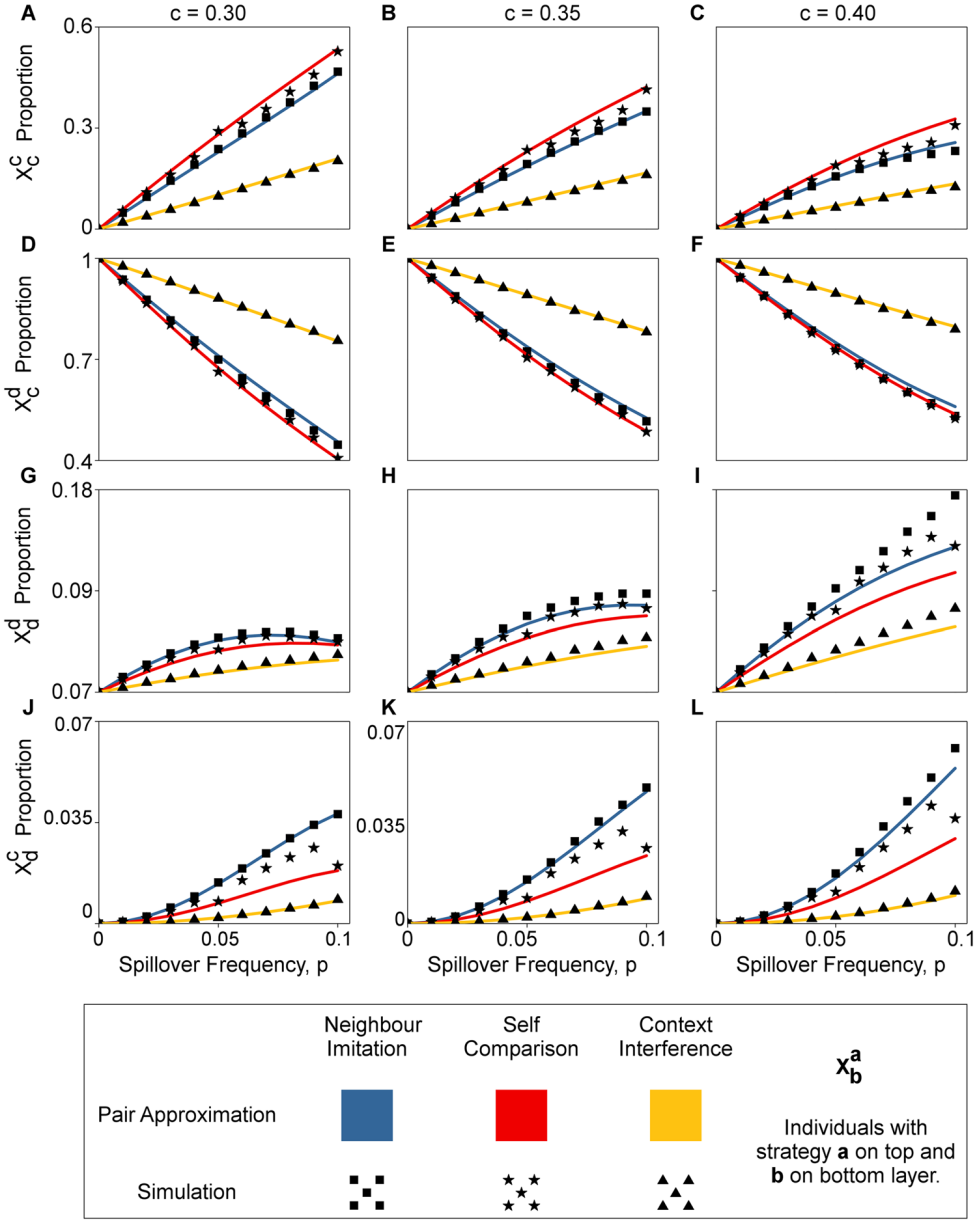
Microscopic characteristics of spillover modes. Figure compares proportion of individual top-bottom strategy combinations of each spillover mode for the parameters in Fig. 2, with different scales on the vertical axes. Each spillover mode differs in their microscopic characteristics. In (**A**–**C**), self comparison has the highest proportion of individuals cooperating on both layers, ${X}_{c}^{c}$. In (**D**–**F**), context interference has the highest proportion with the payoff maximizing strategy profile ${X}_{c}^{d}$. While in (**G**–**L**), neighbour imitation has the largest ${X}_{d}^{d}$ and ${X}_{d}^{c}$ proportions. Parameters: *n* = 3600, *m* = 4, *b* = 1, *β* = 0.2, *α* = 0.5. Simulations: 6 × 10^6^ time steps, averaged over 100 runs. *c* is the cost of cooperation.

Figure 3 further illuminates the differences in microscopic details between the three spillover modes for a subset of the parameter combinations in Fig. 2. Figure 3A–C shows SCS having the highest ${X}_{c}^{c}$, while Fig. 3D–F shows CIS having the highest ${X}_{c}^{d}$. We get a clearer view of how NIS differs from the rest, with higher ${X}_{d}^{d}$ and ${X}_{d}^{c}$ (Fig. 3G–L). Supplementary Figs S4 and S5 extends Fig. 3 for a range of *p* from 0 to 0.40.

**Figure 4:**
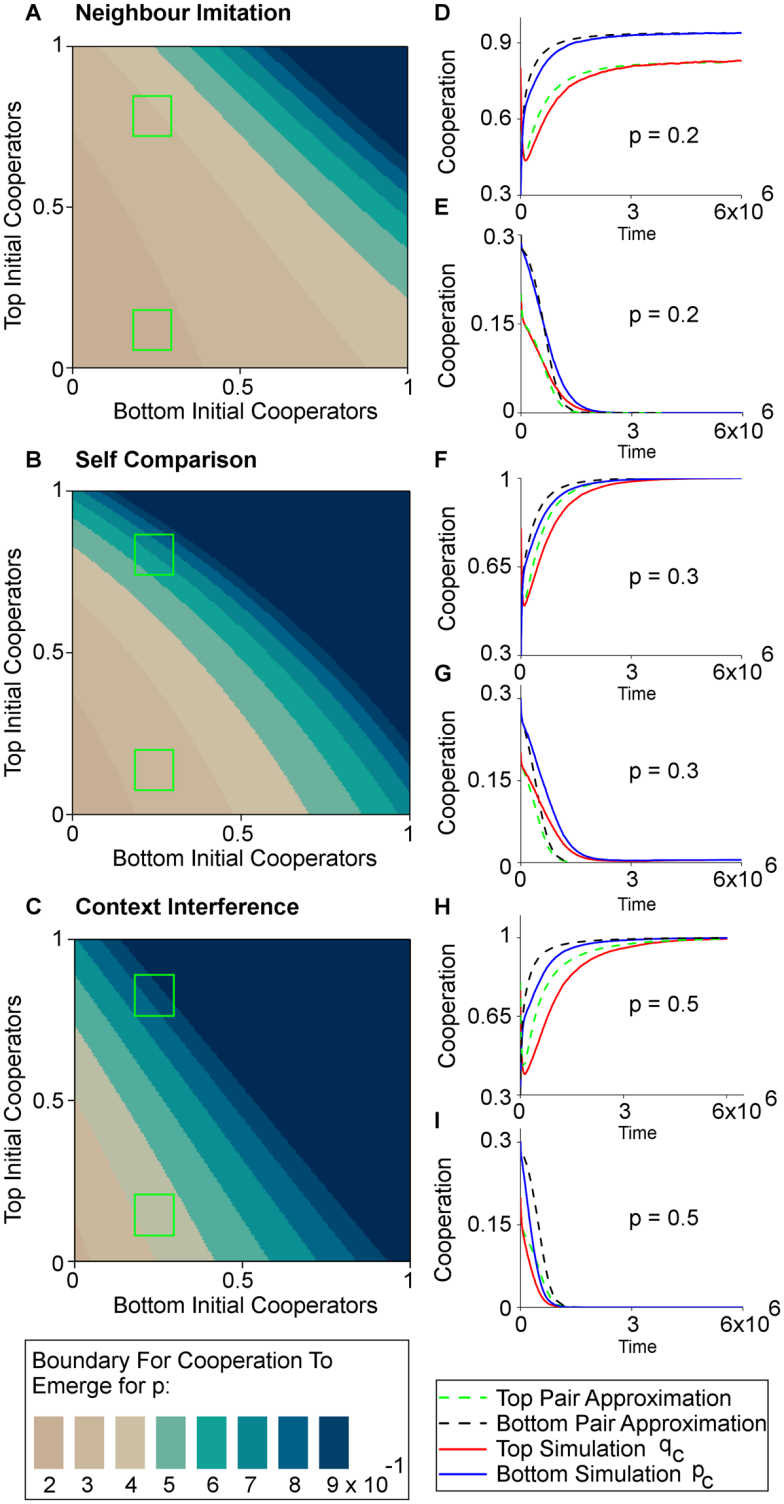
Bistability phenomenon. Spillover can hinder or promote cooperation depending on initial conditions. (**A**–**C**) illustrates, for each frequency of spillover *p*, the initial level of cooperators that would result in zero cooperation at equilibrium, under each spillover mode. In all cases, for a fixed *p*, there is a threshold level of initial cooperators on each network layer beyond which the spillover effect switches to working in favor of cooperation. The initial proportion of cooperators required for spillover effect to give rise to cooperation becomes more demanding as *p* increases. Context interference is shown to be most resilient to this, followed by self comparison, while neighbour imitation is the most vulnerable. (**D**–**I**) demonstrates the bistability phenomenon for selected parameter combinations $(\square )$ with both simulation and pair approximation. Parameters: *n* = 3600, *m* = 4, *c* = 0.35, *b* = 1, *β* = 0.2, *α* = 0.5. Data for (**A**–**C**) starts at 0.05 and ends at 0.95 initial cooperators for both layers. Simulations in (**D**–**I**) 6 × 10^6^ time steps, averaged over 200 runs.

In our previous results, we have initialised individual strategies *C* or *D* on both layers uniformly at random. But how will spillover behave when the initial probability of being a cooperator varies on each layer? We address this question by exhaustively exploring the parameter space using pair approximation as illustrated by Fig. 4 (NIS Fig. 4A, SCS Fig. 4B, CIS Fig. 4C).

Here, we discover a bistability phenomenon. For a fixed frequency of spillover *p*, the parameter space is partitioned into two distinct regions. Depending on the initial proportion of cooperators on each network layer, the spillover effect can either help or hinder cooperation, as shown in more detailed plots by both simulation and pair approximation (Fig. 4D–I). These detailed plots are also reproduced with *p* = 0 in Supplementary Fig. S6 in order to highlight the impact of spillover. This bistability phenomenon has potential social policy implications: if the proportion of cooperators in one setting can be actively raised to a sufficient level, spillover can promote overall levels of cooperation.

As shown in Fig. 4, the number of initial cooperators on the bottom layer has a larger impact on whether spillover hinders or helps cooperation than the number of initial cooperators on the top layer. An example of this can be seen in Fig. 4A, where at *p* = 0.3, when the number of initial cooperators on the top layer is close to zero, sufficient number of initial cooperators on the bottom layer can still result in a spillover effect that helps cooperation. In contrast, for close to zero initial cooperators on the bottom layer, no amount of initial cooperators on the top layer will result in a beneficial effect of spillover on cooperation.

**Figure 5:**
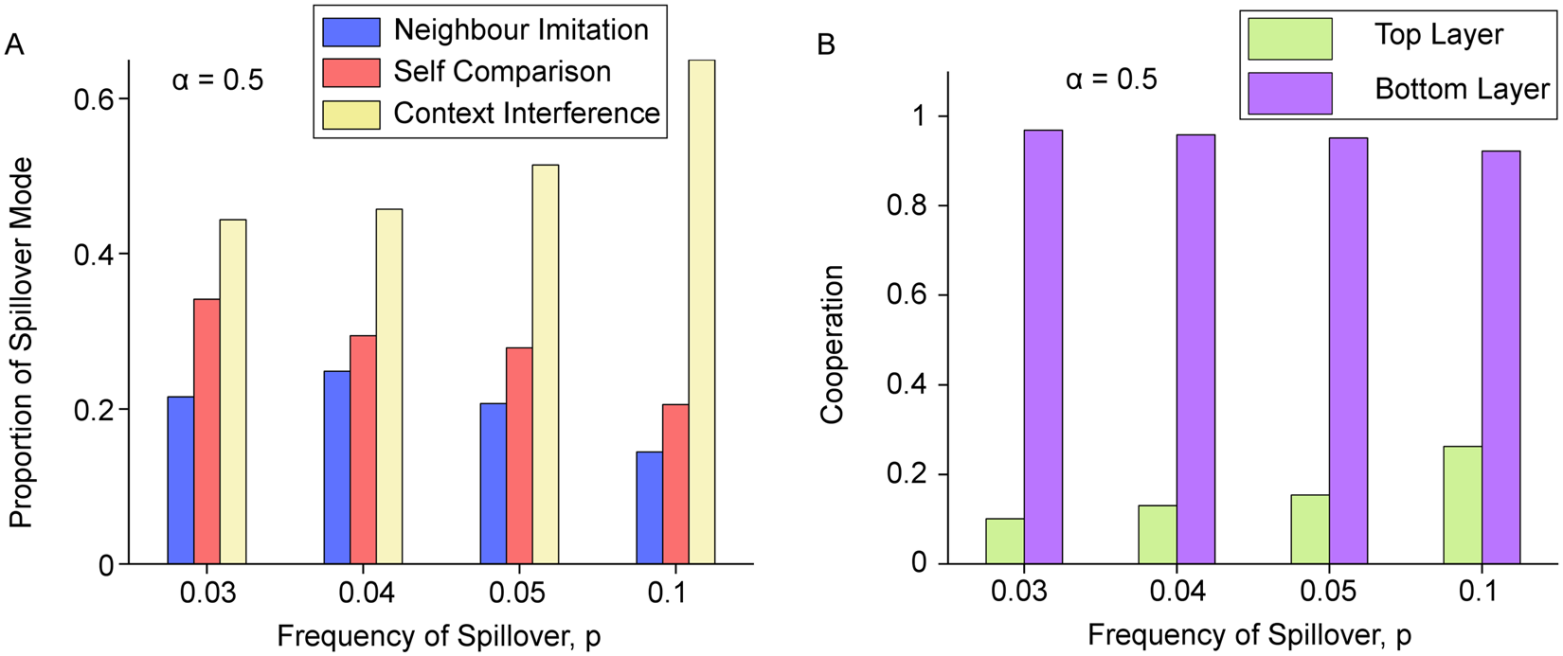
Coevolution of spillover modes and cooperation. Figure 5 shows the proportion of each spillover mode, for low values of *p*, when spillover modes coevolve with strategy in the presence of mutation. Self comparison (red) is second place in (**A**) despite having the most individuals who cooperate on both layers. Context interference (yellow) has the largest proportion due to having the most individuals playing the payoff maximizing combination of C on the bottom layer and D on the top layer. Neighbour imitation (blue) accounts for the least proportion of the population. (**B**) shows that cooperation in the top layer increases with *p* but at the expense of cooperation in the bottom layer. Parameters: *n* = 400, *m* = 4, *c* = 0.35, *β* = 0.2, *b* = 1. Mutation rate *μ* = 10^−4^, 7.2 × 10^9^ total time steps, combined from at most 7 runs for both (**A** and **B**).

So far, we have studied these three spillover modes separately. Next, we will compare them when all three modes are present and are potentially competing with each other. We initialise each individual with a spillover mode uniformly at random and allow the modes to coevolve with strategy while at the same time introducing mutation. Here, the imitation of spillover modes is decided using the sum of payoffs across both layers in order to isolate the effect of natural selection on spillover modes, and to avoid conflating natural selection with cross context interference. Figure 5 shows our results for various *p*.

Each bar in Fig. 5A indicates the proportion of individuals with each of the three spillover mode. For all of the choices of *p* in Fig. 5A, the highest is CIS, followed by SCS and then NIS, with this trend becoming more prominent as *p* increases. We explain the dominance of CIS for this parameter combination by the crucial role of individuals playing *D* on the top prisoner’s dilemma layer, and *C* on the bottom iterated prisoner’s dilemma layer (Fig. 3). When *p* is small, the top layer is approximately at full defection, while the bottom layer is approximately at full cooperation. So, playing *D* on top and *C* on the bottom offers the highest total payoff on average. As we saw in Figs 2F and 3, CIS shows a relative abundance of individuals with this type of mixed strategy, providing a convincing explanation for the supremacy of CIS in the competition.

SCS mode produces a relatively high proportion of individuals cooperating on both layers, which might intuitively be what one would desire in such a social system. However, this tendency results in a loss of individuals playing the optimal combination of strategies (${X}_{c}^{d}$), leading to a loss of competitiveness when pitted against other spillover modes, under the parameter combination of Fig. 5A. The dominance of CIS appears to be robust when the cost of cooperation *c* was lowered to *c* = 0.30, 0.25 and 0.20 (see Supplementary Fig. S7).

However, at *c* = 0.20, this dominance is reduced. Conditions are favourable for cooperation, so we have high equilibrium proportions of cooperators on both layers (Supplementary Fig. S8A). In this situation, ${X}_{c}^{c}$ has a higher payoff than ${X}_{c}^{d}$ and hence, NIS and SCS become more competitive. This increase in competitiveness of the strategy that cooperates on both layers can be overcome by an increase in *p* (Supplementary Fig. S7A for *p* = 0.05). However, this does not happen when cooperation on both levels are high enough, and CIS once again loses its advantage (Supplementary Figs S7A and S8A, show the value *c* = 0.20, *p* = 0.1).

**Figure 6:**
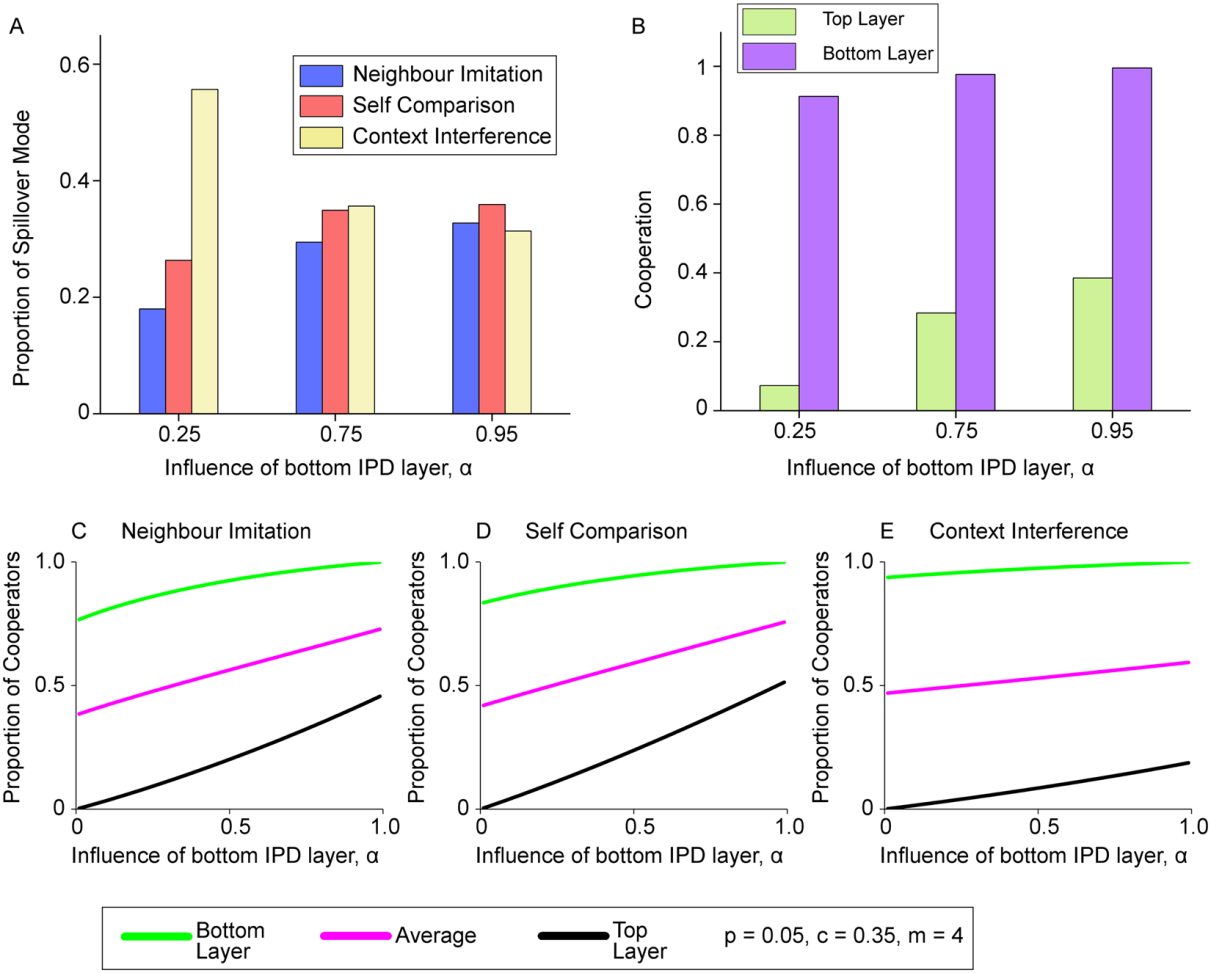
Uneven influence of multiplex layers in spillover process. Figure 6 shows the effect of varying parameter *α* ∈ (0, 1) which governs the relative influence of the two multiplex layers. (**A**) shows that as *α* increases, self comparison spillover mode can have the highest proportion instead. A high *α* allows repeated interactions in the bottom layer to have a large influence and creates more favourable conditions for cooperation in the top layer, as shown by (**B**). This allows for individuals cooperating on both layers, which is plentiful under SCS, to become competitive during coevolution. (**C**–**E**) shows how cooperation in the multiplex increases with *α* under each spillover mode. Context interference is seen to be most resilient against the effect of spillover. Parameters for (**A**,**B**) *n* = 400, *m* = 4, *b* = 1, *c* = 0.35, *β* = 0.2, *p* = 0.05. Mutation rate *μ* = 10^−3^, at least 10^9^ total time steps, combined from at most 10 runs. Parameters for (**C**–**E**) *m* = 4, *b* = 1, *c* = 0.35, *β* = 0.2, *p* = 0.05, generated using pair approximation.

In Fig. 6, we consider an additional parameter *α* ∈ (0, 1) which governs the relative influence of the two layers. When spillover occurs under NIS and SCS, *α* is the probability that an individual on the top layer is chosen to possibly learn a strategy from the bottom layer. Under CIS, *α* is the probability that an individual uses her bottom layer strategy during spillover. We make these definitions so that parameter *α* consistently refers to how strong an influence the bottom IPD layer has when spillover occurs.

We find that higher *α* promotes cooperation in general (Fig. 6C–E) and this could potentially alter the results of coevolution. As shown in Fig. 6A, it is possible for SCS to be favoured by selection instead of CIS when we set *α* = 0.95. This happens because there is a much higher level of cooperation in both layers when *α* is high (Figs 5B, 6B and S9B) which allows ${X}_{c}^{c}$ to be more competitive than ${X}_{c}^{d}$. We show comparison of the cooperation level on both layers over time for both the *α* = 0.5 and *α* = 0.95 scenarios described in Supplementary Figs S10 and S11.

## Discussion

Our results generate testable hypotheses that can inspire future research. Several experiments^21–24^ in the literature had participants play the iterated prisoner’s dilemma and then switch to various versions of one-shot games. All of these cases reported that the iterated prisoner’s dilemma, with conditions that favour cooperation, had a positive effect on cooperation level in the subsequent one-shot game.

Using setups similar to the experiments, one of the spillover mode could be incorporated to test for the existence of an optimal frequency of spillover *p*. For instance, NIS could be implemented experimentally by periodically hiding or revealing the strategies of neighbours on a layer. Similarly, CIS could be implemented by occasionally hiding or mislabelling the layers. At the same time, this can be used to test if spillover modes differ in the proportion of individuals playing each of the four possible top-bottom strategy combinations (Fig. 3), which offers a novel method for comparing and categorizing the myriad of spillover mechanisms that are possible.

Finally, our findings regarding the bistability that arise from varying the initial levels of cooperation on each layer (Fig. 4) could be leveraged to promote cooperation through spillover. As was done in a recent human behaviour experiment^37^, mixing automated bots with human subjects can lead to the desired cooperation level.

## Methods

In our model, *n* individuals are placed on two network layers. The top layer, *T*, is a random regular network of degree four, regenerated with every run, while the bottom layer, *B*, is a two dimensional lattice with periodic boundaries. Individuals play a special version of the prisoner’s dilemma game, known as the donation game, with their neighbours on the top layer: they are initially a cooperator *C* = [1, 0]^*T*^ or defector *D* = [0, 1]^*T*^ with equal probability. The payoff matrix *M*_*T*_ that we use for prisoner’s dilemma is,

1 $${M}_{T}=[\begin{array}{cc}b-c & -c\\ b & 0\end{array}].$$

where *b* is the benefit of cooperation, while *c* is the cost of cooperation. On the bottom layer, individuals play the iterated prisoner’s dilemma game with their partners, where the game is repeated *m* times. They initially start with the strategy *C* or *D* with equal probability. In this case, *C* refers to tit-for-tat (TFT), where the individual cooperates on the first iteration and plays the opponent’s previous strategy for future iterations. Here, *D* stands for always defecting (ALLD) during every iteration. The payoff matrix *M*_*B*_ for this is,

2 $${M}_{B}=[\begin{array}{cc}m(b-c) & -c\\ b & 0\end{array}].$$

Let ${s}_{i}^{k}\,\in \,\{[1,\,{0]}^{T}\mathrm{,}\,[0,{1]}^{T}\}$ be the strategy of individual *i* on layer *k* = {*T*, *B*}, and *M*_*k*_ be the payoff matrix of the game on layer *k* = {*T*, *B*}. Then, the total payoff of an individual *i* on layer *k* is given by,

3 $${P}_{i}^{k}=\sum _{j\,\in \,{{\mathscr{N}}}_{i}^{k}}{({s}_{i}^{k})}^{T}{M}_{k}{s}_{j}^{k},$$

where ${{\mathscr{N}}}_{i}^{k}$ is the neighbourhood of *i* on layer *k*.

At each discrete time step, we randomly choose a focal individual. Under NIS and SCS, with probability 1 − *p*, this individual updates her strategy. Otherwise, with probability *p*, spillover occurs. Spillover results in the transmission of cooperative (C) or defective (D) behaviour across layers, these behaviours correspond to the strategies C or D in both the IPD and PD games. Under CIS, this individual always updates her strategy using a modified procedure described below.

### Strategy updating

If individual *i* chooses to update her strategy, the top layer is chosen as the focal layer with probability $\frac{1}{2}$. Otherwise, the bottom layer is chosen as the focal layer. Next, one of her neighbour *j* on the focal layer is picked at random. Then, the probability that *i* copies the strategy of *j* on the focal layer *k* is given by the Fermi equation^38,39^,

4 $$F({s}_{j}^{k}\to {s}_{i}^{k})=\frac{1}{1+{e}^{-\beta ({P}_{j}^{k}-{P}_{i}^{k})}},$$

where parameter *β* determines the intensity of selection, and ${P}_{i}^{k},{P}_{j}^{k}$ are the total payoffs within the layer *k*, of the focal individual *i* and the neighbour *j* respectively.

### Neighbour Imitation

If spillover occurs under NIS, we choose the top layer to be the focal layer with probability *α*. Otherwise, with probability 1 − *α*, we choose the bottom layer. A neighbour on the layer opposite to the focal layer is chosen randomly. Then, the focal individual does payoff comparison and strategy updating on the non-focal layer. However, if she decides to copy this neighbour’s strategy, the strategy is instead applied to focal layer, as illustrated by Fig. 1B.

### Self Comparison

If spillover occurs under SCS, we choose the top layer to be the focal layer with probability *α*. Otherwise, with probability 1 − *α*, we choose the bottom layer. The focal individual then does payoff comparison and strategy updating with herself on the layer opposite to the focal layer, as shown in Fig. 1C. This means that there is a chance for the individual’s strategy on the focal layer to be replaced with her strategy on the non-focal layer. However, during SCS, her payoff on the bottom IPD layer is normalized by dividing the payoff matrix throughout by the number of game iterations. The payoff matrix used for SCS is,

5 $$\begin{array}{c}\begin{array}{ccccccc} &  &  & C &  &  & D\end{array}\\ \begin{array}{c}C\\ \\ \\ \\ D\end{array}\,(\begin{array}{cc}b-c & -\frac{c}{m}\\ \frac{b}{m} & 0\end{array})\end{array}$$

### Context Interference

Under CIS, individuals always update their strategy. However, the procedure for doing so is modified. During strategy updating, both the focal individual and the randomly chosen neighbour independently has a probability *p* of experiencing context interference. If one of them does, she has an independent probability *α* of using her bottom layer strategy for all parts of the strategy updating procedure. Otherwise, with probability 1 − *α*, she uses her top layer strategy. These definitions are made so that parameter *α* consistently refers to how strong an influence the bottom IPD layer has when spillover occurs. This modified strategy updating procedure is shown in Fig. 1D.

### Coevolution and mutation

Figure 5 was generated by subjecting the three spillover modes to co-evolution and mutation. Individuals initially are assigned a spillover mode at random. We then proceed with two distinct phases: the regular phase and subsequently the coevolution phase. During the regular phase, at each discrete time step, a focal individual is chosen at random, then according to her spillover mode, strategy updating or spillover is carried out as described above. During the coevolution phase, a focal individual and a focal layer are chosen uniformly at random. The focal individual then picks a neighbour on the focal layer at random. Their total payoffs on the multiplex network is then calculated by summing up their total payoffs across both layers. Payoff comparison is then done using the Fermi equation, which gives the probability of the focal individual copying the neighbour’s spillover mode. These two phases are run for 10^4^ time steps each, and then repeated in the same order (regular then coevolution phase) until the desired total number of time steps is achieved when summed across all phases.

In both the regular and coevolution phases, whenever strategy update is successful, there is a probability *μ* of mutation. When mutation occurs, strategy or spillover mode is selected at random, instead of copied.

### Analytical solutions

We derived analytical solutions for each of the spillover mode, taking into account population structure, using extended pair approximation. We refer readers to the SI for the pair approximation equations and their details.

### Data availability

The datasets and code supporting this article have been uploaded as part of the supplementary material.

## Electronic supplementary material

Supplementary Information
