# Supplementary material for: Spillover modes in multiplex games: double-edged effects on cooperation and their coevolution
Source: Sci Rep. 2018 May 2;8:6922. doi: 10.1038/s41598-018-25025-3 (PMC5932083; doi:10.1038/s41598-018-25025-3)
Supplement: Supplementary file 1 — Supplementary Information [file 41598_2018_25025_MOESM1_ESM.pdf]

Spillover modes in multiplex games: double-edged effects on cooperation, and their coevolution

Tommy Khoo, Feng Fu, Scott Pauls

Supplementary Information

List of Figures

1 Cooperation under highly beneficial or detrimental conditions. . . . 2
2 Iterated prisoner's dilemma (IPD) game on both layers. . . . 3
3 Prisoner's dilemma (PD) game on both layers. . . . 4
4 Extended Fig. 3 for X_c^c and X_c^d. . . . 5
5 Extended Fig. 3 for X_d^d and X_d^c. . . . 6
6 Plot of Fig. 4D-I without spillover ($p = 0$). . . . 7
7 Coevolution of spillover modes for $c = 0.30, 0.25, 0.20$. . . . 8
8 Fine details for spillover modes at $c = 0.20$ and 0.30. . . . 9
9 Coevolution of spillover modes for $\alpha = 0.95$. . . . 10
10 Time evolution of cooperation and spillover modes for $\alpha = 0.95, c = 0.35$ case. . . . 11
11 Time evolution of cooperation and spillover modes for $\alpha = 0.50, c = 0.35$ case. . . . 12

Contents

1 Datasets and Code 13

2 Pair Approximation 13

2.0.1 Inter-layer Notation 13
2.0.2 Top Layer Notation 13
2.0.3 Bottom Layer Notation 13
2.0.4 Top Layer Transition Probabilities Notation and Definitions 13
2.0.5 Bottom Layer Transition Probabilities Notation and Definitions 14
2.0.6 Notation for Probabilities of Specific Events and Configurations 15
2.1 Neighbour Imitation Spillover 15
2.2 Self Comparison Spillover 19
2.3 Context Interference Spillover 21

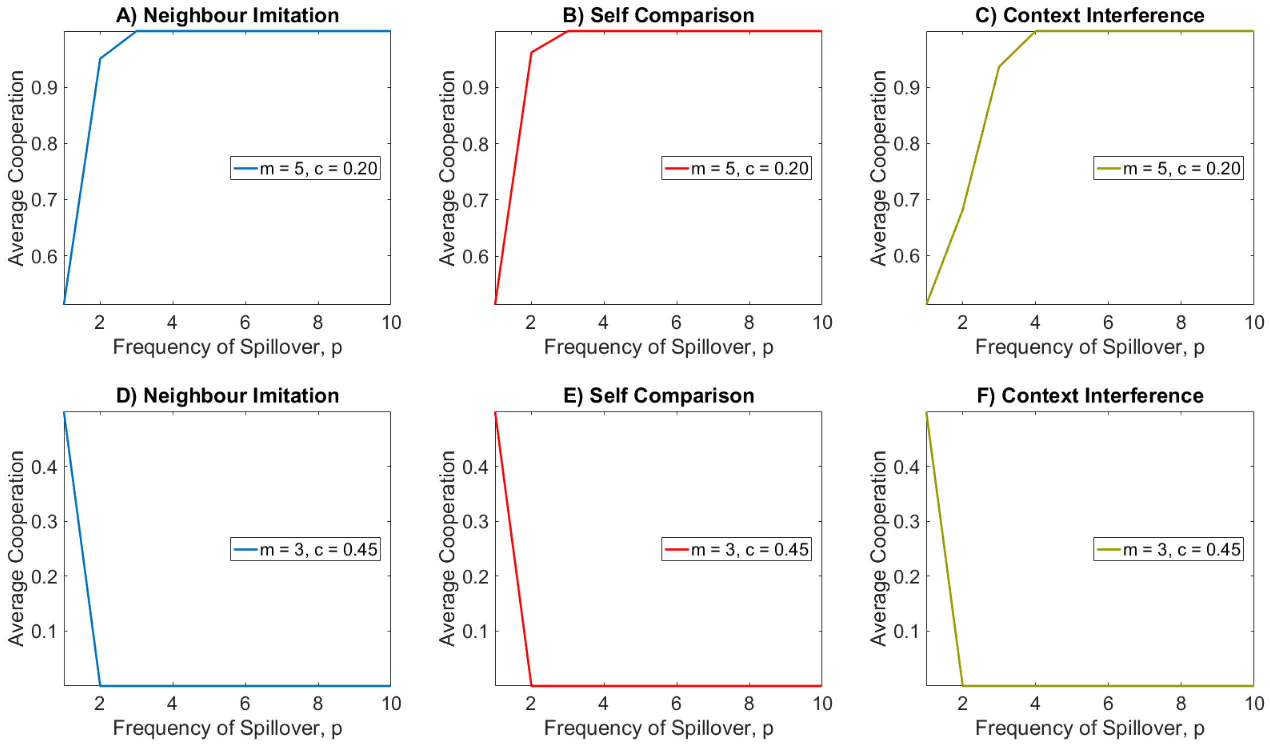

Figure S 1. Cooperation under highly beneficial or detrimental conditions. Figure indicates that when conditions are highly beneficial or detrimental to cooperation, average cooperation level in the multiplex network rapidly rise to one or decline to zero monotonically. As illustrated by Fig. 2 in the main text, an optimal p arise for conditions in between these extremes. Parameters: $\beta = 0.2$, $b = 1$, $\alpha = 0.5$. Results generated using pair approximation.

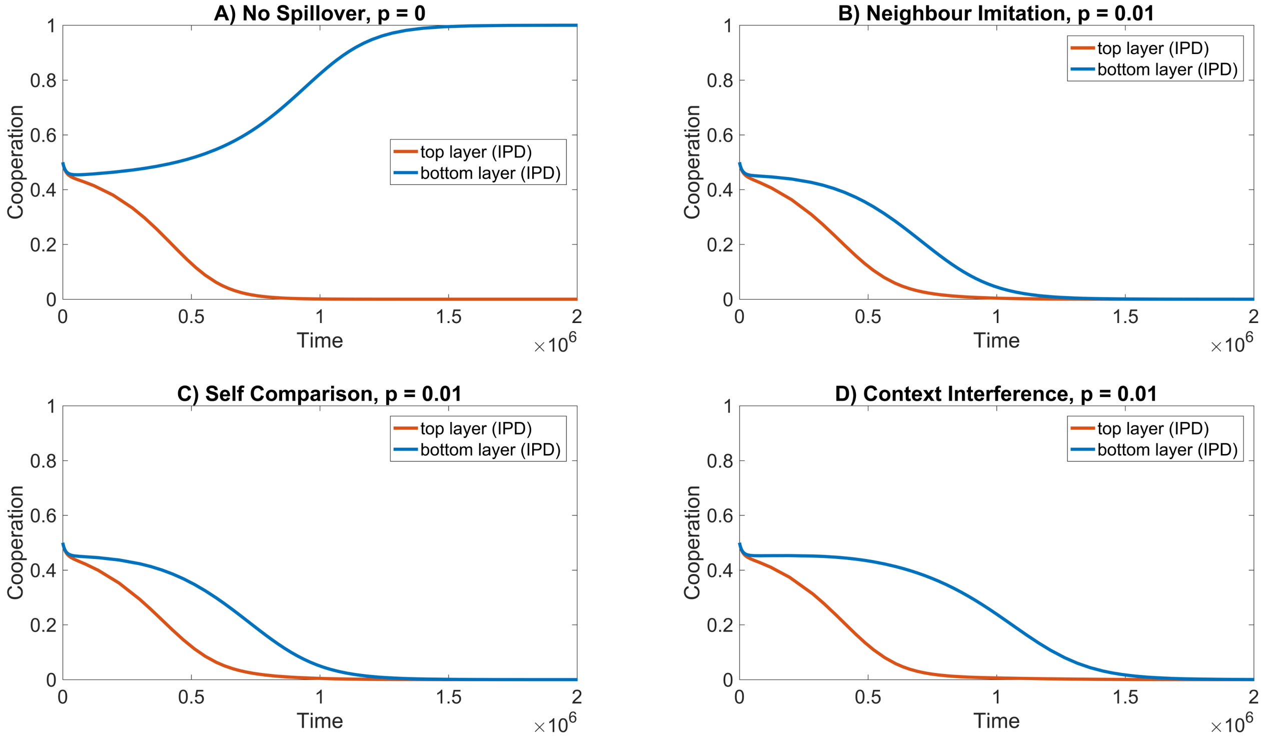

Figure S 2. Iterated prisoner's dilemma (IPD) game on both layers. Figure shows the case in which the same IPD game is played on both layers, with a parameter choice that allows the bottom layer to reach full cooperation at equilibrium, while cooperation in the top layer goes to zero. Introducing a small amount of spillover $p = 0.01$ results in the average cooperation level declining to zero under all three spillover modes. Conditions are not conducive enough for cooperation on the bottom layer, which is overwhelmed by the detrimental conditions in the top layer. Context interference can be observed to be more resistant to spillover. Parameters: $n = 3600$, $\beta = 0.2$, $m = 3$, $b = 1$, $c = 0.50$, $\alpha = 0.5$ for both layers. Results generated using pair approximation.

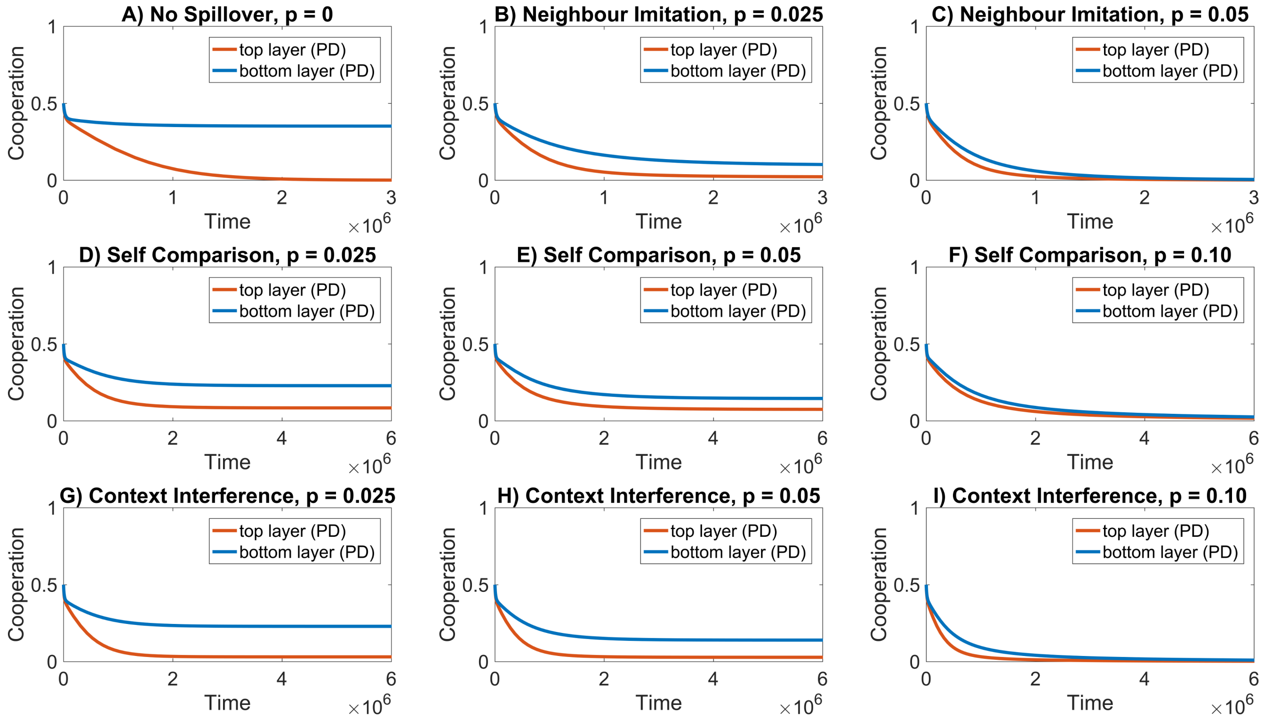

Figure S 3. Prisoner's dilemma (PD) game on both layers. Figure shows the case in which the same PD game is played on both layers. Parameters are chosen so that cooperators can survive in the bottom layer at equilibrium, while cooperation in the top layer goes to zero. As spillover is increased from $p = 0$ to $p = 0.10$, average cooperation in the multiplex goes to zero for all three spillover modes. While the lattice structure in the bottom layer allowed some cooperation to survive, this effect is not strong enough to overcome the detrimental conditions in the top layer. Neighbour imitation is observed to be most susceptible to the influence of spillover. Parameters: $n = 3600$, $\beta = 0.2$, $b = 3$, $c = 0.05$, $\alpha = 0.5$ for both layers. Results generated using pair approximation.

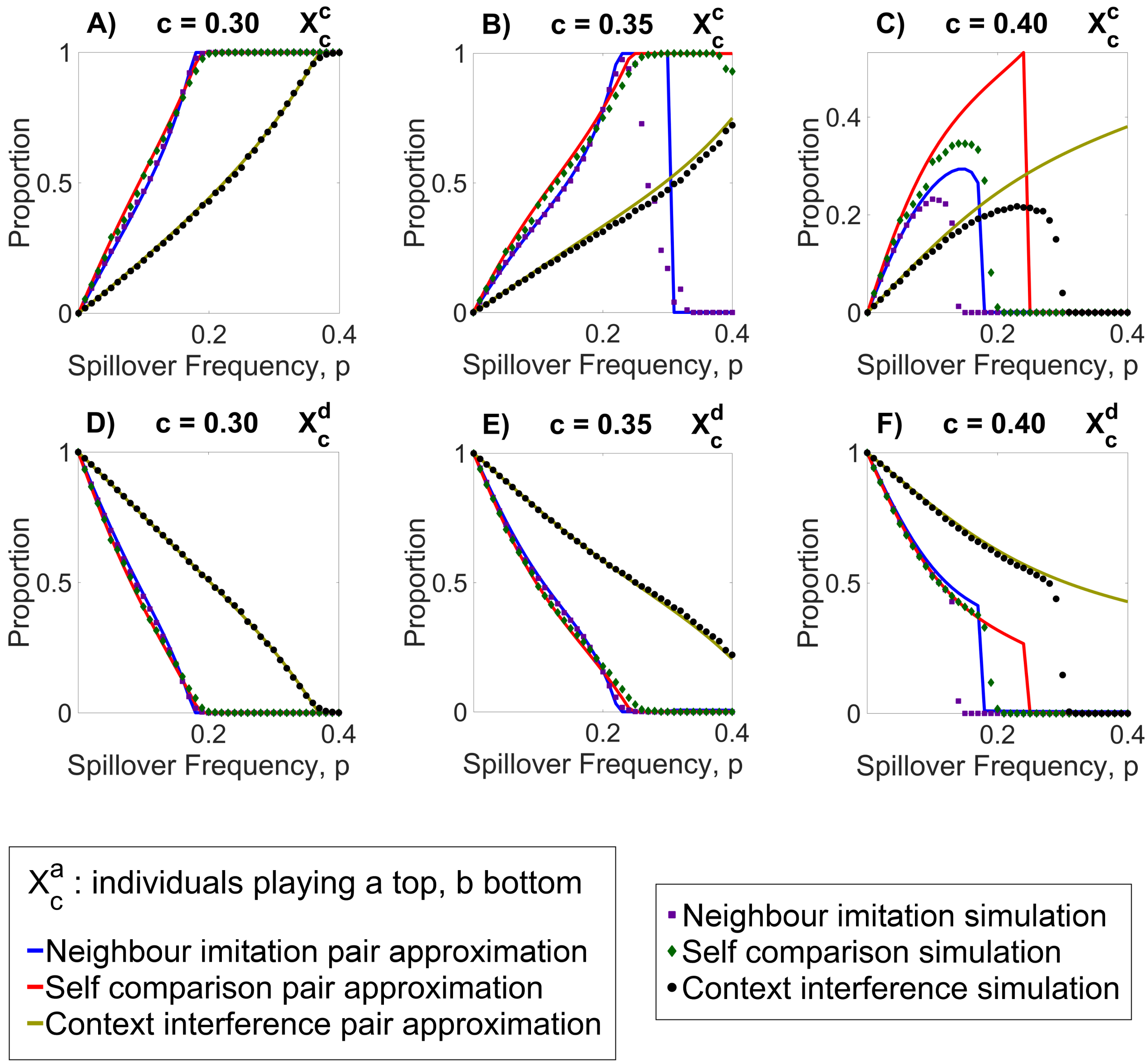

Figure S 4. **Extended Fig. 3 for X_c^c and X_c^d.** Figure shows an extended version of Fig. 3 in the main text for the proportion of individuals with strategy profile X_c^c and X_c^d respectively, for each spillover mode. Parameters: $n = 3600$, $b = 1$, $\beta = 0.2$, $\alpha = 0.5$. Parameter p range from $p = 0$ to $p = 0.40$ in steps of 0.01. c is the cost of cooperation. Simulations: 6×10^6 time steps, averaged over 100 runs.

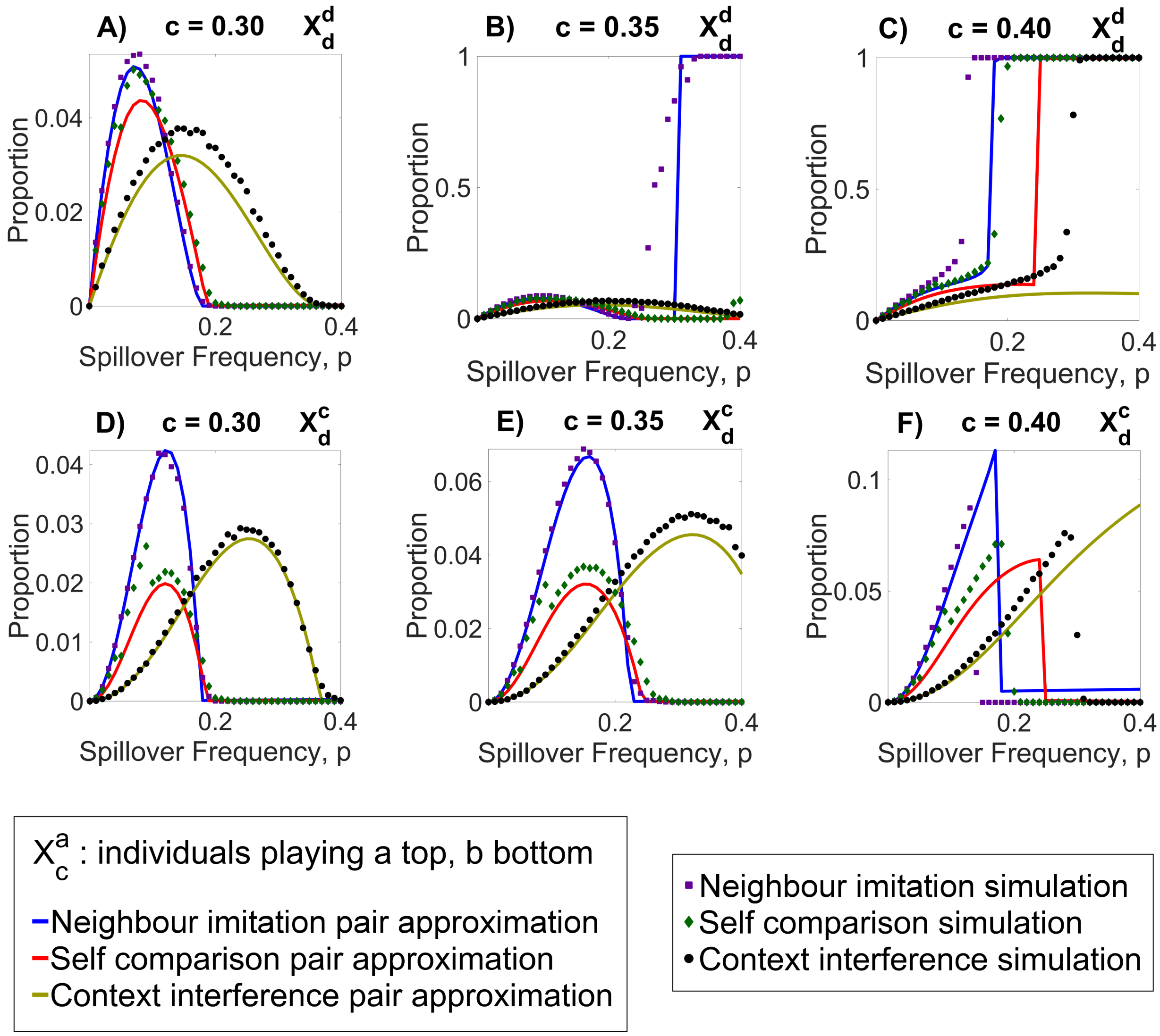

Figure S 5. **Extended Fig. 3 for X_d^d and X_d^c.** Figure shows an extended version of Fig. 3 in the main text for the proportion of individuals with strategy profile X_d^d and X_d^c respectively, for each spillover mode. Parameters: $n = 3600$, $b = 1$, $\beta = 0.2$, $\alpha = 0.5$. Parameter p range from $p = 0$ to $p = 0.40$ in steps of 0.01. c is the cost of cooperation. Simulations: 6×10^6 time steps, averaged over 100 runs.

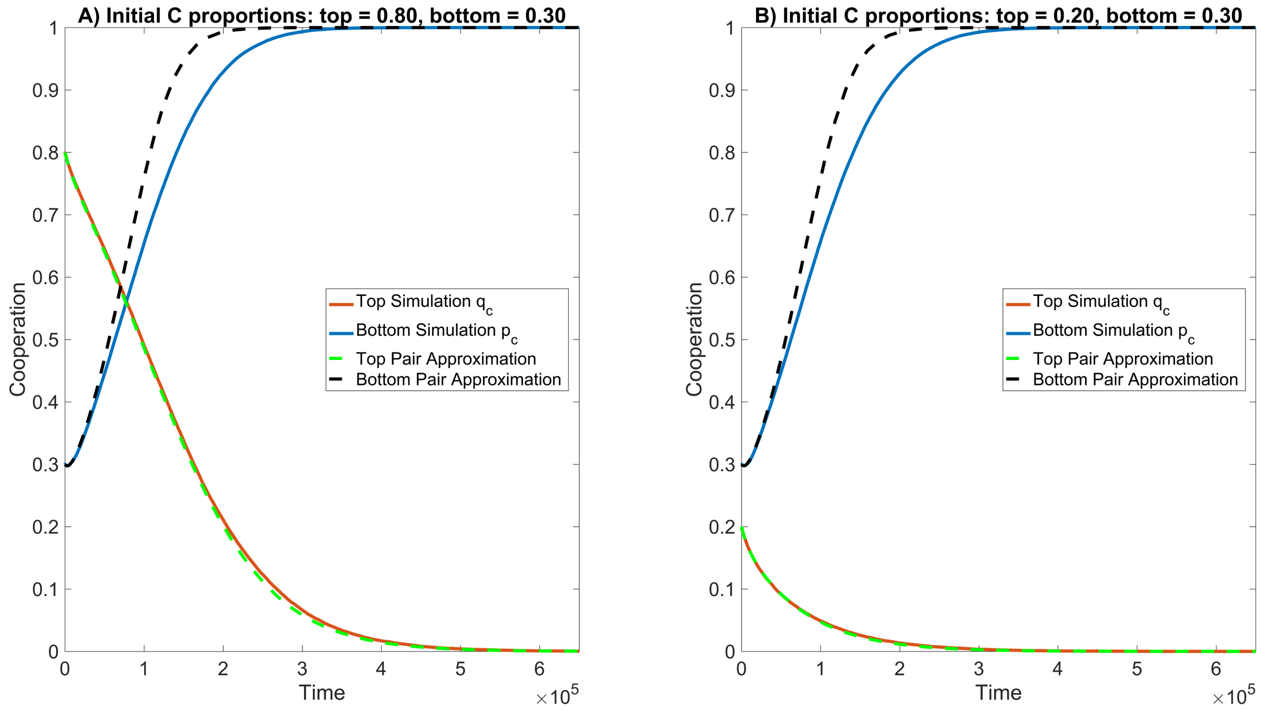

Figure S 6. **Plot of Fig. 4D-I without spillover ($p = 0$).** Figures show simulation and pair approximation results, with no spillover ($p = 0$), for the parameters and initial proportions of cooperators in Fig. 4D-I of the main text. Without spillover, the bottom IPD layer goes to full cooperation while the top PD layer goes to zero cooperation. Parameters: $n = 3600$, $m = 4$, $b = 1$, $c = 0.35$, $\beta = 0.2$, $\alpha = 0.5$, Simulations: 6×10^6 time steps, averaged over 100 runs. Truncated to 6.5×10^5 time steps in the figure.

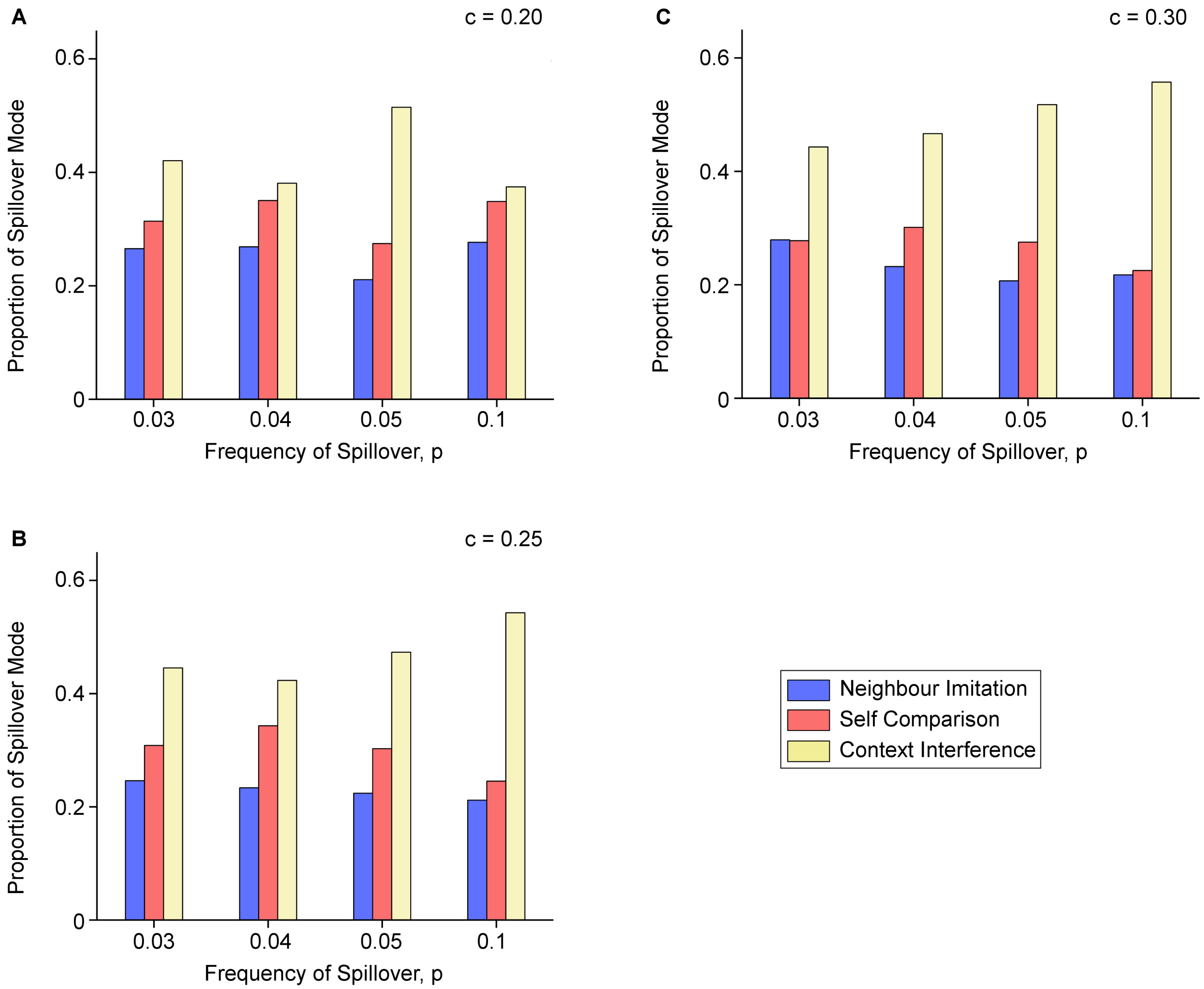

Figure S 7. Coevolution of spillover modes for $c = 0.30, 0.25, 0.20$**.** Proportion of spillover modes under coevolution and mutation are shown here for parameters different from Fig. 5A in the main text. For cost of cooperation $c = 0.30$, $p = 0.03$, we see that neighbour imitation and self comparison are eventually matched, but the competitive advantage of self comparison is exaggerated with a higher $p = 0.04$. At $c = 0.20$, when conditions are very favourable for cooperation, context interference starts to lose its lead. However, if support for cooperation is not strong enough, context interference can still maintain its lead with a sufficiently high p, as shown by the $c = 0.20$, $p = 0.05$ case. Fig. S5 contain more details explaining these phenomena. Parameters: $n = 400$, $\beta = 0.2$, $b = 1$, $\alpha = 0.5$, mutation rate $\mu = 10^{-4}$. Simulation results consists of at least 4.7×10^9 total time steps, combined over 6 runs.

A c = 0.20 case

p = 0.03	q_c	p_c	X_c^c	X_c^d
**NIS**	0.2249	0.9728	0.2193	0.7535
**SCS**	0.2784	0.9811	0.2757	0.7054
**CIS**	0.0998	0.9925	0.0990	0.8935

p = 0.04	q_c	p_c	X_c^c	X_c^d
**NIS**	0.3056	0.9670	0.2964	0.6706
**SCS**	0.3704	0.9771	0.3660	0.6110
**CIS**	0.1349	0.9902	0.1335	0.8567

p = 0.05	q_c	p_c	X_c^c	X_c^d
**NIS**	0.3892	0.9633	0.3763	0.5870
**SCS**	0.4615	0.9746	0.4555	0.5191
**CIS**	0.1709	0.9882	0.1689	0.8193

p = 0.1	q_c	p_c	X_c^c	X_c^d
**NIS**	0.8653	0.9843	0.8535	0.1308
**SCS**	0.9053	0.9904	0.9011	0.0893
**CIS**	0.3651	0.9811	0.3583	0.6228

B c = 0.30 case

p = 0.03	q_c	p_c	X_c^c	X_c^d
**NIS**	0.1433	0.9612	0.1380	0.8233
**SCS**	0.1751	0.9717	0.1724	0.7993
**CIS**	0.0612	0.9881	0.0604	0.9277

p = 0.04	q_c	p_c	X_c^c	X_c^d
**NIS**	0.1927	0.9500	0.1836	0.7664
**SCS**	0.2323	0.9635	0.2275	0.7359
**CIS**	0.0824	0.9843	0.0811	0.9032

q_c : Proportion of top cooperators.

p_c : Proportion of bottom cooperators.

X_b^a : Proportion of individuals playing top **a** bottom **b**.

NIS : Neighbour Imitation Spillover

SCS : Self Comparison Spillover

CIS : Context Interference Spillover

Figure S 8. **Fine details for spillover modes at** $c = 0.20$ **and** 0.30. Figure shows, for each spillover mode, the proportion of cooperators q_c, p_c on each network layer, as well as the strategy profiles X_c^c, X_c^d. Fig. S5A explains what we see with the $c = 0.20$ case in Fig. S4A. As p is increased from 0.03 to 0.04, we see a rise in cooperation in both network layers, leading to an increase in the competitiveness of X_c^c and the resulting decline in context interference spillover mode. This effect can be suppressed with a higher $p = 0.05$ while cooperation level remains similar. However, as cooperation levels increase dramatically at $p = 0.1$, context interference once again loses its advantage. Parameters: $n = 400$, $m = 4$, $b = 1$, $\beta = 0.2$, $\alpha = 0.5$. Results generated by pair approximation.

A

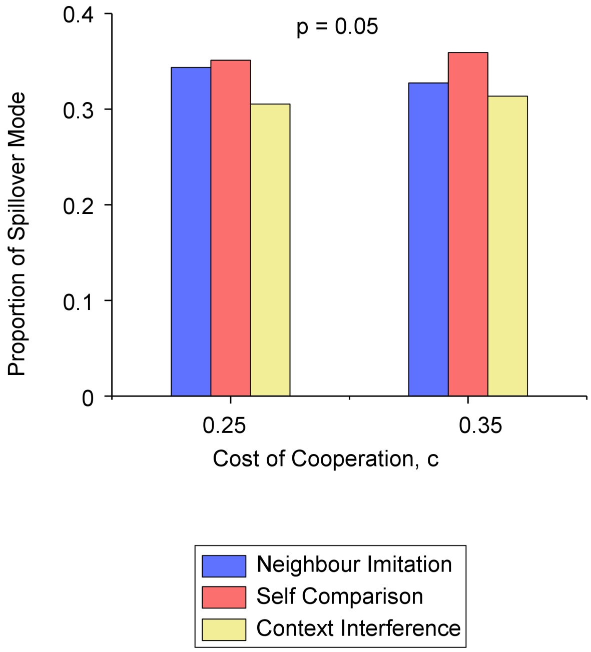

B

**c = 0.25**	**q**$_c$	**p**$_c$	$\mathbf{X}_c^c$	$\mathbf{X}_c^d$
**NIS**	0.6407	0.9976	0.6393	0.3583
**SCS**	0.7138	0.9984	0.7132	0.2852
**CIS**	0.2717	0.9988	0.2714	0.7274

**c = 0.35**	**q**$_c$	**p**$_c$	$\mathbf{X}_c^c$	$\mathbf{X}_c^d$
**NIS**	0.4333	0.9950	0.4312	0.5638
**SCS**	0.4895	0.9962	0.4885	0.5078
**CIS**	0.1781	0.9979	0.1777	0.8202

q_c :Proportion of top cooperators.

p_c : Proportion of bottom cooperators.

X_b^a : Proportion of individuals playing top **a** bottom **b**.

NIS : Neighbour Imitation Spillover

SCS : Self Comparison Spillover

CIS : Context Interference Spillover

Figure S 9. **Coevolution of spillover modes for** $\alpha = 0.95$**.** Figure shows how a high $\alpha = 0.95$ can change which spillover mode takes the lead in the coevolution and mutation case. Parameter α is the probability, during spillover, of selecting the bottom repeated local interaction layer to influence the top one-shot layer. When repeated local interactions have a much higher influence, conditions are much more favourable for cooperation on both network layers. The strategy profile X_c^c can become more competitive when compared to X_c^d. Parameters: $n = 400$. $\alpha = 0.95$, $m = 4, b = 1$, $\beta = 0.2$, mutation rate $\mu = 10^{-3}$. Simulation in Fig. S6A has at least 1.4×10^9 time steps, combined over 6 runs. Fig. S6B produced using pair approximation.

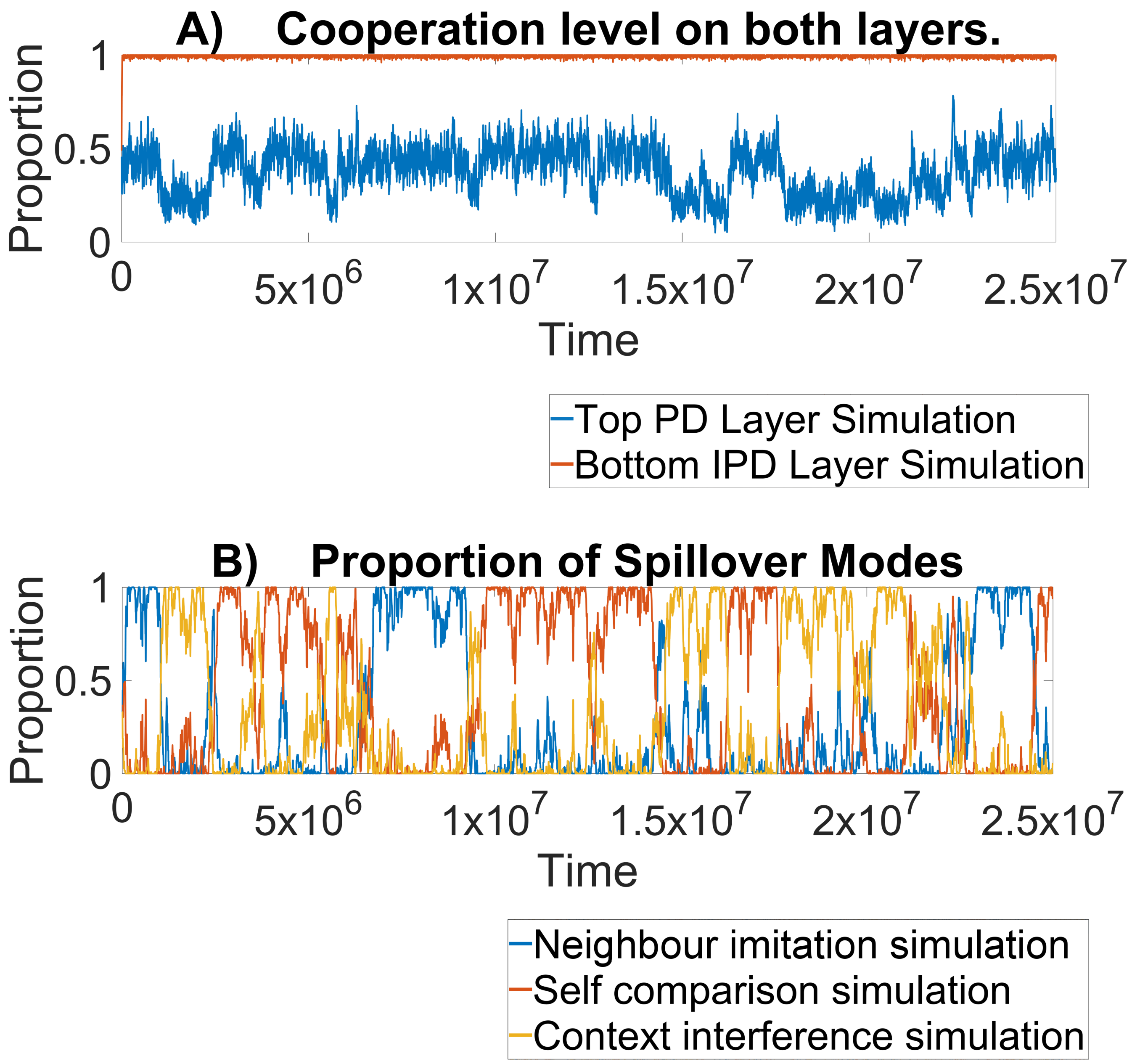

Figure S 10. Time evolution of cooperation and spillover modes for $\alpha = 0.95, c = 0.35$ case. Figure demonstrates how neighbour imitation (NIS) and self comparison (SCS) produces a higher level of cooperation in the multiplex when they are dominant, and how context interference (CIS) results in a lower level of cooperation when it is dominant. This lead to NIS and SCS becoming much harder to invade by CIS as X_c^c now has higher competitiveness. Parameters: $n = 400$, $m = 4$, $b = 1$, $\beta = 0.2$, $p = 0.05$, $\alpha = 0.95$, $c = 0.35$. Mutation rate $\mu = 10^{-3}$. Simulation data taken from the first 2.5×10^7 time steps of a single run.

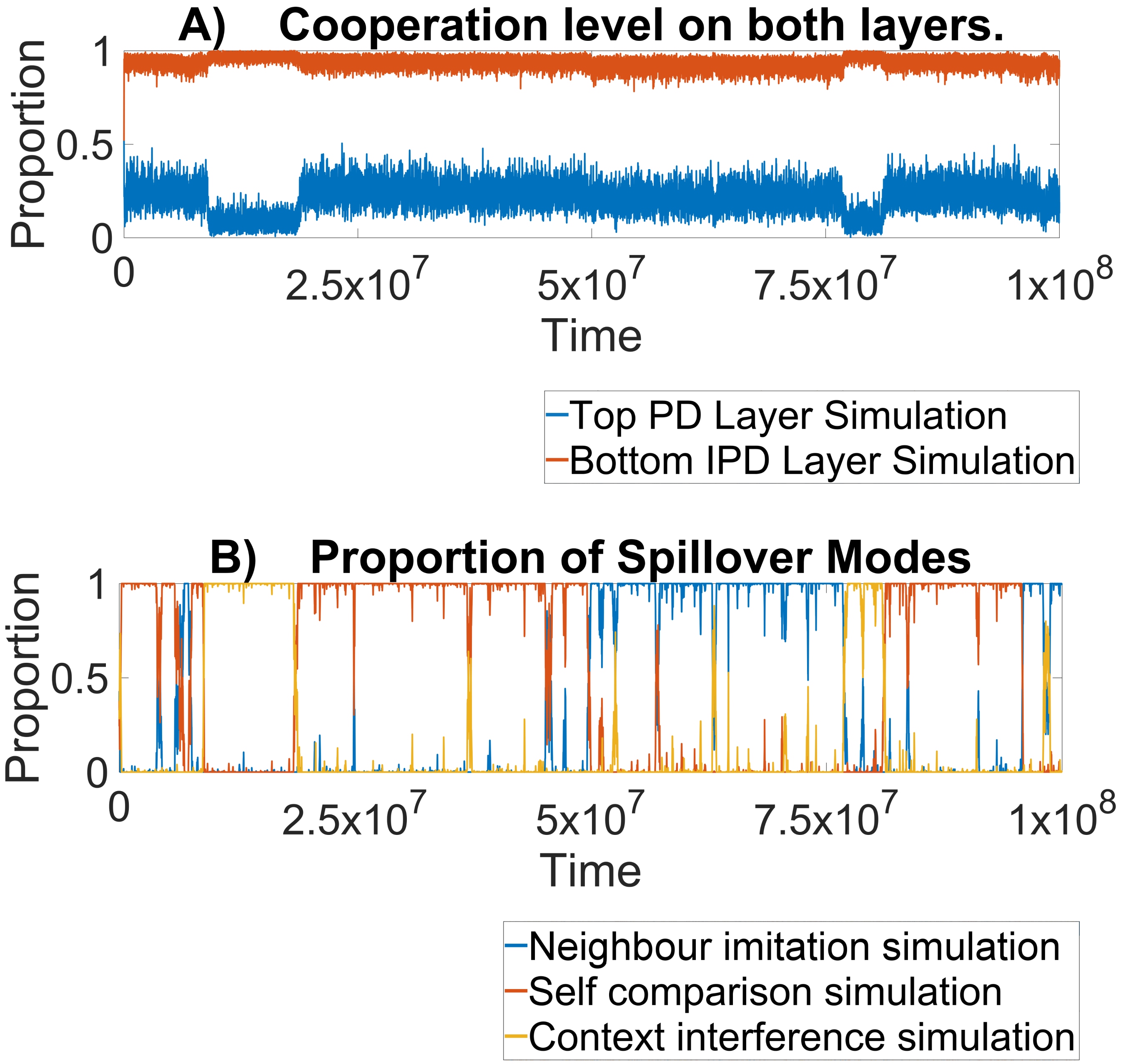

Figure S 11. Time evolution of cooperation and spillover modes for $\alpha = 0.50, c = 0.35$ case. Figure illustrates results for a parameter combination from Fig. 5 in the main text. Here, we see that cooperation levels are at a lower level than in Fig. S7. Thus, X_c^d is much more competitive than X_c^c, leading to the dominance of context interference spillover mode. Parameters: $n = 400$, $m = 4$, $b = 1$, $\beta = 0.2$, $p = 0.05$, $\alpha = 0.95$, $c = 0.35$. Mutation rate $\mu = 10^{-4}$. Simulation data taken from the first 1×10^8 time steps of a single run.

1 Datasets and Code

Datasets and code supporting this article: Figshare doi:10.6084/m9.figshare.5480548

2 Pair Approximation

We will use pair approximation to derive analytic solutions to each of the three spillover modes. Our analytic solution for each mode consists of differential equations for $\dot{q}_c, \dot{p}_c, \dot{q}_{cc}, \dot{p}_{cc}, \dot{x}^c_c$ and $\dot{x}^d_c$. We start with the notation and definitions common to all three spillover modes.

2.0.1 Inter-layer Notation

Let x^a_b be the fraction of individuals with strategies a on the top prisoner's dilemma layer, and b on the bottom iterated prisoner's dilemma layer. Letting c be cooperate and d defect, we have the four cases: x^c_c, x^d_c, x^c_d and x^d_d, as well as $x^c_c + x^d_c + x^c_d + x^d_d = 1$.

2.0.2 Top Layer Notation

Let q_c and q_d be the top layer fraction of C and D respectively. We must have $q_c + q_d = 1$. Let q_{ab} be the top layer fraction of connected pairs with strategies a and b. We have four cases q_{cc}, q_{cd}, q_{dc} and q_{dd}, as well as $q_{cc} + q_{cd} + q_{dc} + q_{dd} = 1$. Let the conditional probabilities be $q_{c|c} = \frac{q_{cc}}{q_c}$, $q_{d|c} = \frac{q_{dc}}{q_c}$, $q_{c|d} = \frac{q_{cd}}{q_d}$, and $q_{d|d} = \frac{q_{dd}}{q_d}$. We must have $q_{c|c} + q_{d|c} = 1$, and $q_{d|d} + q_{c|d} = 1$.

2.0.3 Bottom Layer Notation

Let p_c and p_d be the bottom layer fraction of C and D respectively. We must have $p_c + p_d = 1$. Let p_{ab} be the bottom layer fraction of connected pairs with strategies a and b. We have four cases p_{cc}, p_{cd}, p_{dc} and p_{dd}, as well as $p_{cc} + p_{cd} + p_{dc} + p_{dd} = 1$. Let the conditional probabilities be $p_{c|c} = \frac{p_{cc}}{p_c}$, $p_{d|c} = \frac{p_{dc}}{p_c}$, $p_{c|d} = \frac{p_{cd}}{p_d}$, and $p_{d|d} = \frac{p_{dd}}{p_d}$. We must have $p_{c|c} + p_{d|c} = 1$, and $p_{d|d} + p_{c|d} = 1$.

2.0.4 Top Layer Transition Probabilities Notation and Definitions

The probability of strategy b replacing strategy a is given by the Fermi equation,

$$F(\pi_b, \pi_a) = \frac{1}{1 + e^{-\beta(\pi_b - \pi_a)}},$$

where π_j is the average payoff from using strategy j. Let T be the top layer and B the bottom layer. Let the payoff matrix for layer $i \in \{T, B\}$ be,

$$\begin{pmatrix} R_i & S_i \\ T_i & P_i \end{pmatrix}.$$

Let k_i be the average degree of individuals in layer $i \in \{T, B\}$. Finally, let $W_{b \to a}$ be the probability that, if two individuals on the top layer are chosen for strategy updating, the event "strategy b replaces a" occurs. Then, we have

$$W_{c \to c} = W_{d \to d} = \frac{1}{2}.$$

For $W_{c \to d}$ and $W_{d \to c}$, we need to consider the average payoff of a pair of connected individuals with different strategies. So, we have

$$\pi_c = (k_T - 1) q_{c|c} \cdot R_T + (1 + (k_T - 1) q_{d|c}) \cdot S_T,$$

$$\pi_d = (1 + (k_T - 1)q_{c|d}) \cdot T_T + (k_T - 1)q_{d|d} \cdot P_T,$$

$$W_{c\to d} = F(\pi_c, \pi_d),$$

$$W_{d\to c} = F(\pi_d, \pi_c).$$

2.0.5 Bottom Layer Transition Probabilities Notation and Definitions

Let a pair of connected individuals with strategies A and B be an AB pair. Consider a CC pair in the bottom layer, which is a two dimensional lattice with periodic boundaries. We call the individual that is selected for strategy updating the focal individual, while the randomly chosen neighbour the non-focal individual. Let x, y, z be the strategies of the three other neighbours of the focal individual. Let u, v, w be the strategies of the three other neighbours of the non-focal individual.

Let $n(x, y, z)$ be the number of strategy C among the strategies x, y, z. Then, the average payoff of the focal individual is now

$$\pi_c = (n(x, y, z) + 1) \cdot R_B + (3 - n(x, y, z)) \cdot S_B.$$

The average payoff of the non-focal individual is,

$$\pi_c' = (n(u, v, w) + 1) \cdot R_B + (3 - n(u, v, w)) \cdot S_B.$$

Let $\phi_{b\to a}$ be the probability that, if the focal individual is using strategy a and the non-focal individual is using strategy b, strategy b is sucessful at replacing strategy a. Putting it all together, the probability that a focal C copies a neighbouring non-focal C in the bottom layer is,

$$\phi_{c\to c} = \frac{p_{cx}p_{cy}p_{cz}p_{cu}p_{cv}p_{cw}}{p_c^3 p_c^3} F(\pi_c', \pi_c),$$

we used the fact that $p_{x|c}p_{y|c}p_{z|c} = \frac{p_{cx}p_{cy}p_{cz}p_{cu}p_{cv}p_{cw}}{p_c^3 p_c^3}$.

For a DD pair in the bottom layer, the average payoff of the focal node and non-focal node are respectively,

$$\pi_d = n(x, y, z) \cdot T_B + (1 + (3 - n(x, y, z))) \cdot P_B,$$

$$\pi_d' = n(u, v, w) \cdot T_B + (1 + (3 - n(u, v, w))) \cdot P_B.$$

Then, the probability that a focal D copies a neighbouring non-focal D in the bottom layer is,

$$\phi_{d\to d} = \frac{p_{dx}p_{dy}p_{dz}p_{du}p_{dv}p_{dw}}{p_d^3 p_d^3} F(\pi_d^{'}, \pi_d).$$

Finally, we consider CD pairs. The average payoffs are,

$$\pi_c = n(x, y, z) \cdot R_B + (1 + (3 - n(x, y, z))) \cdot S_B,$$

$$\pi_d = (1 + n(u, v, w)) \cdot T_B + (3 - n(u, v, w)) \cdot P_B.$$

And then the probability that a focal C copies a neighbouring non-focal D in the bottom layer is,

$$\phi_{d\to c} = \frac{p_{cx}p_{cy}p_{cz}p_{du}p_{dv}p_{dw}}{p_c^3 p_d^3} F(\pi_d, \pi_c).$$

Also, for DC pairs. The average payoffs are,

$$\pi_d = (1 + n(x, y, z)) \cdot T_B + (3 - n(x, y, z)) \cdot P_B.$$

$$\pi_c = n(u, v, w) \cdot R_B + (1 + (3 - n(u, v, w))) \cdot S_B,$$

The probability that a focal D copies a neighbouring non-focal C in the bottom layer is,

$$\phi_{c \to d} = \frac{p_{dx} p_{dy} p_{dz} p_{cu} p_{cv} p_{cw}}{p_d^3 p_c^3} F(\pi_c, \pi_d).$$

2.0.6 Notation for Probabilities of Specific Events and Configurations

In each of the three spillover modes, there are certain configurations of individuals and strategies that can lead to a change in the system. Let x, y, z be strategies. Let the focal node have top strategy x and bottom strategy z. Let the non-focal neighbour node have top strategy y and bottom strategy w. Then, we represent the event that the top strategy x is replaced with strategy y by,

$$\begin{array}{ccc} \text{x} \longleftarrow \text{y} & & \text{(top layer)} \\ \Big| \qquad \Big| & & \\ \text{z} \qquad \text{w} & & \text{(bottom layer)} \end{array} .$$

If the bottom strategies are not specified then the event includes all combinations of strategies on the bottom. For example, the event that the top strategy x is replaced with strategy y, regardless of strategies on the bottom, is represented by,

$$\begin{array}{ccc} \text{x} \longleftarrow \text{y} & & \text{(top layer)} \\ \Big| \qquad \Big| & & \\ & & \text{(bottom layer)} \end{array} .$$

For the context interference spillover mode, if an individual with strategy x is using strategy v due to context interference, we denote her strategy as $x[v]$. Let the probability of an event E be $\mathbb{P}(E)$. We are also using the facts that for the top layer, $x_c^c + x_d^c = q_c$, $x_d^d + x_c^d = q_d$, and that for the bottom layer, $x_c^c + x_c^d = p_c$, $x_d^d + x_d^c = p_d$. Finally, we let $\mathbb{E}(E)$ be the expected number of change in $C - C$ links when event E occurs.

2.1 Neighbour Imitation Spillover

At each discrete time step, a random individual is chosen. With probability p, the chosen individual does inter-layer neighbour imitation spillover updating. Otherwise, with probability $1 - p$, she does intra-layer strategy updating. During neighbour imitation spillover updating, the top layer is chosen as the focal layer to **receive** spillover strategies with probability α. Otherwise, with probability $1 - \alpha$ the bottom layer will be chosen as the focal layer to **receive** spillover strategies from the top.

Then, the individual's strategy on the chosen focal layer might be replaced by the strategy of a random neighbour on the **opposite** non-focal layer. Payoff comparison between this neighbour and the individual is then done, also on the opposite non-focal layer. This neighbour's strategy has a chance of being adopted by the individual on focal layer, given by the Fermi equation. Fig. 1B in the main text illustrates this process. We list the probabilities of all related events and configurations below.

$$\mathbb{P}\left(\begin{array}{l} \text{C} \longleftarrow \text{D} \\ \Big| \\ \text{C} \end{array}\right) = (1-p) \cdot \frac{1}{2} \cdot q_c \cdot q_{d|c} \cdot W_{d \to c} \cdot \frac{x_c^c}{q_c}, \qquad \mathbb{P}\left(\begin{array}{l} \text{C} \longleftarrow \text{D} \\ \Big| \\ \text{D} \end{array}\right) = (1-p) \cdot \frac{1}{2} \cdot q_c \cdot q_{d|c} \cdot W_{d \to c} \cdot \frac{x_d^c}{q_c}.$$

$$\mathbb{P}\left(\begin{array}{ccc} D & \longleftarrow & C \\ | & & \\ C & & \end{array}\right) = (1-p)\cdot\frac{1}{2}\cdot q_d\cdot q_{c|d}\cdot W_{c\to d}\cdot\frac{x_c^d}{q_d},\qquad \mathbb{P}\left(\begin{array}{ccc} D & \longleftarrow & C \\ | & & \\ D & & \end{array}\right) = (1-p)\cdot\frac{1}{2}\cdot q_d\cdot q_{c|d}\cdot W_{c\to d}\cdot\frac{x_d^d}{q_d}.$$

$$\mathbb{P}\left(\begin{array}{ccc} C & & \\ | & & \\ C & \longleftarrow & D \end{array}\right) = (1-p)\cdot\frac{1}{2}\cdot p_c\cdot p_{d|c}\cdot \phi_{d\to c}\cdot\frac{x_c^c}{p_c},\qquad \mathbb{P}\left(\begin{array}{ccc} D & & \\ | & & \\ C & \longleftarrow & D \end{array}\right) = (1-p)\cdot\frac{1}{2}\cdot p_c\cdot p_{d|c}\cdot \phi_{d\to c}\cdot\frac{x_c^d}{p_c}.$$

$$\mathbb{P}\left(\begin{array}{ccc} C & & \\ | & & \\ D & \longleftarrow & C \end{array}\right) = (1-p)\cdot\frac{1}{2}\cdot p_d\cdot p_{c|d}\cdot \phi_{c\to d}\cdot\frac{x_d^c}{p_d},\qquad \mathbb{P}\left(\begin{array}{ccc} D & & \\ | & & \\ D & \longleftarrow & C \end{array}\right) = (1-p)\cdot\frac{1}{2}\cdot p_d\cdot p_{c|d}\cdot \phi_{c\to d}\cdot\frac{x_d^d}{p_d}.$$

$$\mathbb{P}\left(\begin{array}{ccc} C & — & D \\ | & \swarrow & \\ C & & \end{array}\right) = p\cdot(1-\alpha)\cdot q_c\cdot q_{d|c}\cdot W_{d\to c}\cdot\frac{x_c^c}{q_c},\qquad \mathbb{P}\left(\begin{array}{ccc} C & — & C \\ | & \swarrow & \\ D & & \end{array}\right) = p\cdot(1-\alpha)\cdot q_c\cdot q_{c|c}\cdot W_{c\to c}\cdot\frac{x_d^c}{q_c}.$$

$$\mathbb{P}\left(\begin{array}{ccc} D & — & D \\ | & \swarrow & \\ C & & \end{array}\right) = p\cdot(1-\alpha)\cdot q_d\cdot q_{d|d}\cdot W_{d\to d}\cdot\frac{x_c^d}{q_d},\qquad \mathbb{P}\left(\begin{array}{ccc} D & — & C \\ | & \swarrow & \\ D & & \end{array}\right) = p\cdot(1-\alpha)\cdot q_d\cdot q_{c|d}\cdot W_{c\to d}\cdot\frac{x_d^d}{q_d}.$$

$$\mathbb{P}\left(\begin{array}{ccc} C & & \\ | & \nwarrow & \\ C & — & D \end{array}\right) = p\cdot\alpha\cdot p_c\cdot p_{d|c}\cdot \phi_{d\to c}\cdot\frac{x_c^c}{p_c},\qquad \mathbb{P}\left(\begin{array}{ccc} C & & \\ | & \nwarrow & \\ D & — & D \end{array}\right) = p\cdot\alpha\cdot p_d\cdot p_{d|d}\cdot \phi_{d\to d}\cdot\frac{x_d^c}{p_d}.$$

$$\mathbb{P}\left(\begin{array}{ccc} D & & \\ | & \nwarrow & \\ D & — & C \end{array}\right) = p\cdot\alpha\cdot p_d\cdot p_{c|d}\cdot \phi_{c\to d}\cdot\frac{x_d^d}{p_d},\qquad \mathbb{P}\left(\begin{array}{ccc} D & & \\ | & \nwarrow & \\ C & — & C \end{array}\right) = p\cdot\alpha\cdot p_c\cdot p_{c|c}\cdot \phi_{c\to c}\cdot\frac{x_c^d}{p_c}.$$

Let $n(x, y, z)$ be the number of C strategies among the neighbours of the focal individual. Let k_T be the average degree on the top layer, and k_B be the average degree on the bottom layer. The list of expected change in number of $C - C$ links for relevant events and configurations are as follows.

$$\mathbb{E}\left(\begin{array}{ccc} C & \longleftarrow & D \\ | & & \\ C & & \end{array}\right) = -(k_T-1)q_{c|c},\qquad \mathbb{E}\left(\begin{array}{ccc} C & \longleftarrow & D \\ | & & \\ D & & \end{array}\right) = -(k_T-1)q_{c|c}.$$

$$\mathbb{E}\left(\begin{array}{ccc} D & \longleftarrow & C \\ | & & \\ C & & \end{array}\right) = 1+(k_T-1)q_{c|d},\qquad \mathbb{E}\left(\begin{array}{ccc} D & \longleftarrow & C \\ | & & \\ D & & \end{array}\right) = 1+(k_T-1)q_{c|d}.$$

$$\mathbb{E}\left(\begin{array}{ccc} C & & \\ | & & \\ C & \longleftarrow & D \end{array}\right) = -n(x,y,z),\qquad \mathbb{E}\left(\begin{array}{ccc} D & & \\ | & & \\ C & \longleftarrow & D \end{array}\right) = -n(x,y,z),$$

$$\mathbb{E}\left(\begin{array}{ccc} \mathrm{C} & & \\ | & & \\ \mathrm{D} & \longleftarrow & \mathrm{C} \end{array}\right) = 1 + n(x,y,z), \qquad \mathbb{E}\left(\begin{array}{ccc} \mathrm{D} & & \\ | & & \\ \mathrm{D} & \longleftarrow & \mathrm{C} \end{array}\right) = 1 + n(x,y,z).$$

$$\mathbb{E}\left(\begin{array}{ccc} \mathrm{C} & - & \mathrm{D} \\ | & \swarrow & \\ \mathrm{C} & & \end{array}\right) = -k_B \cdot p_{c|c}, \qquad \mathbb{E}\left(\begin{array}{ccc} \mathrm{C} & - & \mathrm{C} \\ | & \swarrow & \\ \mathrm{D} & & \end{array}\right) = k_B \cdot p_{c|d}.$$

$$\mathbb{E}\left(\begin{array}{ccc} \mathrm{D} & - & \mathrm{D} \\ | & \swarrow & \\ \mathrm{C} & & \end{array}\right) = -k_B \cdot p_{c|c}, \qquad \mathbb{E}\left(\begin{array}{ccc} \mathrm{D} & - & \mathrm{C} \\ | & \swarrow & \\ \mathrm{D} & & \end{array}\right) = k_B \cdot p_{c|d}.$$

$$\mathbb{E}\left(\begin{array}{ccc} \mathrm{C} & & \\ | & \nwarrow & \\ \mathrm{C} & - & \mathrm{D} \end{array}\right) = -k_T \cdot q_{c|c}, \qquad \mathbb{E}\left(\begin{array}{ccc} \mathrm{C} & & \\ | & \nwarrow & \\ \mathrm{D} & - & \mathrm{D} \end{array}\right) = -k_T \cdot q_{c|c}.$$

$$\mathbb{E}\left(\begin{array}{ccc} \mathrm{D} & & \\ | & \nwarrow & \\ \mathrm{D} & - & \mathrm{C} \end{array}\right) = k_T \cdot q_{c|d}, \qquad \mathbb{E}\left(\begin{array}{ccc} \mathrm{D} & & \\ | & \nwarrow & \\ \mathrm{C} & - & \mathrm{C} \end{array}\right) = k_T \cdot q_{c|d}.$$

Let x, y, z be the strategies of the neighbours of the focal individual on the bottom layer. Let u, v, w be strategies of the neighbours of the non-focal individual on the bottom layer. Then, the differential equations for the neighbour influence spillover mode are as follows.

$$\begin{aligned} \dot{q}_c = {} & \mathbb{P}\left(\begin{array}{ccc} \mathrm{D} & \longleftarrow & \mathrm{C} \\ | & & \\ \mathrm{C} & & \end{array}\right) + \mathbb{P}\left(\begin{array}{ccc} \mathrm{D} & \longleftarrow & \mathrm{C} \\ | & & \\ \mathrm{D} & & \end{array}\right) \\ & + \sum_{x,y,z,u,v,w} \left[\mathbb{P}\left(\begin{array}{ccc} \mathrm{D} & & \\ | & \nwarrow & \\ \mathrm{C} & - & \mathrm{C} \end{array}\right) + \mathbb{P}\left(\begin{array}{ccc} \mathrm{D} & & \\ | & \nwarrow & \\ \mathrm{D} & - & \mathrm{C} \end{array}\right) \right] \\ & - \mathbb{P}\left(\begin{array}{ccc} \mathrm{C} & \longleftarrow & \mathrm{D} \\ | & & \\ \mathrm{C} & & \end{array}\right) - \mathbb{P}\left(\begin{array}{ccc} \mathrm{C} & \longleftarrow & \mathrm{D} \\ | & & \\ \mathrm{D} & & \end{array}\right) \\ & - \sum_{x,y,z,u,v,w} \left[\mathbb{P}\left(\begin{array}{ccc} \mathrm{C} & & \\ | & \nwarrow & \\ \mathrm{C} & - & \mathrm{D} \end{array}\right) + \mathbb{P}\left(\begin{array}{ccc} \mathrm{C} & & \\ | & \nwarrow & \\ \mathrm{D} & - & \mathrm{D} \end{array}\right) \right]. \end{aligned}$$

$$\begin{aligned} \dot{q}_{cc} = \frac{2}{k_T} \cdot \Bigg\{ & \mathbb{E}\left(\begin{array}{ccc} \mathrm{C} & \longleftarrow & \mathrm{D} \\ | & & \\ \mathrm{C} & & \end{array}\right) \cdot \mathbb{P}\left(\begin{array}{ccc} \mathrm{C} & \longleftarrow & \mathrm{D} \\ | & & \\ \mathrm{C} & & \end{array}\right) + \mathbb{E}\left(\begin{array}{ccc} \mathrm{C} & \longleftarrow & \mathrm{D} \\ | & & \\ \mathrm{D} & & \end{array}\right) \cdot \mathbb{P}\left(\begin{array}{ccc} \mathrm{C} & \longleftarrow & \mathrm{D} \\ | & & \\ \mathrm{D} & & \end{array}\right) \\ & + \mathbb{E}\left(\begin{array}{ccc} \mathrm{D} & \longleftarrow & \mathrm{C} \\ | & & \\ \mathrm{C} & & \end{array}\right) \cdot \mathbb{P}\left(\begin{array}{ccc} \mathrm{D} & \longleftarrow & \mathrm{C} \\ | & & \\ \mathrm{C} & & \end{array}\right) + \mathbb{E}\left(\begin{array}{ccc} \mathrm{D} & \longleftarrow & \mathrm{C} \\ | & & \\ \mathrm{D} & & \end{array}\right) \cdot \mathbb{P}\left(\begin{array}{ccc} \mathrm{D} & \longleftarrow & \mathrm{C} \\ | & & \\ \mathrm{D} & & \end{array}\right) \end{aligned}$$

$$
+ \sum_{x,y,z,u,v,w} \left[\mathbb{E}\left(\begin{array}{ccc} C & & \\ | & \nwarrow & \\ C & \text{—} & D \end{array} \right) \cdot \mathbb{P}\left(\begin{array}{ccc} C & & \\ | & \nwarrow & \\ C & \text{—} & D \end{array} \right) + \mathbb{E}\left(\begin{array}{ccc} C & & \\ | & \nwarrow & \\ D & \text{—} & D \end{array} \right) \cdot \mathbb{P}\left(\begin{array}{ccc} C & & \\ | & \nwarrow & \\ D & \text{—} & D \end{array} \right) \right.
$$

$$
\left. + \; \mathbb{E}\left(\begin{array}{ccc} D & & \\ | & \nwarrow & \\ C & \text{—} & C \end{array} \right) \cdot \mathbb{P}\left(\begin{array}{ccc} D & & \\ | & \nwarrow & \\ C & \text{—} & C \end{array} \right) + \mathbb{E}\left(\begin{array}{ccc} D & & \\ | & \nwarrow & \\ D & \text{—} & C \end{array} \right) \cdot \mathbb{P}\left(\begin{array}{ccc} D & & \\ | & \nwarrow & \\ D & \text{—} & C \end{array} \right) \right] \Bigg\}
$$

$$
\dot{p}_c = \sum_{x,y,z,u,v,w} \left[\mathbb{P}\left(\begin{array}{ccc} C & & \\ | & & \\ D & \longleftarrow & C \end{array} \right) + \mathbb{P}\left(\begin{array}{ccc} D & & \\ | & & \\ D & \longleftarrow & C \end{array} \right) \right] + \mathbb{P}\left(\begin{array}{ccc} C & \text{—} & C \\ | & \swarrow & \\ D & & \end{array} \right) + \mathbb{P}\left(\begin{array}{ccc} D & \text{—} & C \\ | & \swarrow & \\ D & & \end{array} \right)
$$

$$
- \sum_{x,y,z,u,v,w} \left[\; \mathbb{P}\left(\begin{array}{ccc} C & & \\ | & & \\ C & \longleftarrow & D \end{array} \right) + \mathbb{P}\left(\begin{array}{ccc} D & & \\ | & & \\ C & \longleftarrow & D \end{array} \right) \right] - \mathbb{P}\left(\begin{array}{ccc} C & \text{—} & D \\ | & \swarrow & \\ C & & \end{array} \right) - \mathbb{P}\left(\begin{array}{ccc} D & \text{—} & D \\ | & \swarrow & \\ C & & \end{array} \right).
$$

$$
\dot{p}_{cc} = \frac{2}{k_B} \cdot \Bigg\{ \sum_{x,y,z,u,v,w} \left[\mathbb{E}\left(\begin{array}{ccc} D & & \\ | & & \\ C & \longleftarrow & D \end{array} \right) \cdot \mathbb{P}\left(\begin{array}{ccc} D & & \\ | & & \\ C & \longleftarrow & D \end{array} \right) + \mathbb{E}\left(\begin{array}{ccc} C & & \\ | & & \\ C & \longleftarrow & D \end{array} \right) \cdot \mathbb{P}\left(\begin{array}{ccc} C & & \\ | & & \\ C & \longleftarrow & D \end{array} \right) \right.
$$

$$
\left. + \; \mathbb{E}\left(\begin{array}{ccc} C & & \\ | & & \\ D & \longleftarrow & C \end{array} \right) \cdot \mathbb{P}\left(\begin{array}{ccc} C & & \\ | & & \\ D & \longleftarrow & C \end{array} \right) + \mathbb{E}\left(\begin{array}{ccc} D & & \\ | & & \\ D & \longleftarrow & C \end{array} \right) \cdot \mathbb{P}\left(\begin{array}{ccc} D & & \\ | & & \\ D & \longleftarrow & C \end{array} \right) \right] + \mathbb{E}\left(\begin{array}{ccc} C & \text{—} & D \\ | & \swarrow & \\ C & & \end{array} \right) \cdot \mathbb{P}\left(\begin{array}{ccc} C & \text{—} & D \\ | & \swarrow & \\ C & & \end{array} \right)
$$

$$
+ \; \mathbb{E}\left(\begin{array}{ccc} D & \text{—} & D \\ | & \swarrow & \\ C & & \end{array} \right) \cdot \mathbb{P}\left(\begin{array}{ccc} D & \text{—} & D \\ | & \swarrow & \\ C & & \end{array} \right) + \mathbb{E}\left(\begin{array}{ccc} C & \text{—} & C \\ | & \swarrow & \\ D & & \end{array} \right) \cdot \mathbb{P}\left(\begin{array}{ccc} C & \text{—} & C \\ | & \swarrow & \\ D & & \end{array} \right) + \mathbb{E}\left(\begin{array}{ccc} D & \text{—} & C \\ | & \swarrow & \\ D & & \end{array} \right) \cdot \mathbb{P}\left(\begin{array}{ccc} D & \text{—} & C \\ | & \swarrow & \\ D & & \end{array} \right) \Bigg\}
$$

$$
\dot{x}_c^c = \sum_{x,y,z,u,v,w} \left[\mathbb{P}\left(\begin{array}{ccc} C & & \\ | & & \\ D & \longleftarrow & C \end{array} \right) + \mathbb{P}\left(\begin{array}{ccc} D & & \\ | & \nwarrow & \\ C & \text{—} & C \end{array} \right) \right]
$$

$$
+ \; \mathbb{P}\left(\begin{array}{ccc} D & \longleftarrow & C \\ | & & \\ C & & \end{array} \right) + \mathbb{P}\left(\begin{array}{ccc} C & \text{—} & C \\ | & \swarrow & \\ D & & \end{array} \right) - \mathbb{P}\left(\begin{array}{ccc} C & \longleftarrow & D \\ | & & \\ C & & \end{array} \right) - \mathbb{P}\left(\begin{array}{ccc} C & \text{—} & D \\ | & \swarrow & \\ C & & \end{array} \right)
$$

$$
- \sum_{x,y,z,u,v,w} \left[\mathbb{P}\left(\begin{array}{ccc} C & & \\ | & & \\ C & \longleftarrow & D \end{array} \right) + \mathbb{P}\left(\begin{array}{ccc} C & & \\ | & \nwarrow & \\ C & \text{—} & D \end{array} \right) \right].
$$

$$
\dot{x}_c^d = \sum_{x,y,z,u,v,w} \left[\mathbb{P}\left(\begin{array}{ccc} D & & \\ | & & \\ D & \longleftarrow & C \end{array} \right) + \mathbb{P}\left(\begin{array}{ccc} C & & \\ | & \nwarrow & \\ C & \text{—} & D \end{array} \right) \right]
$$

$$+\ \mathbb{P}\left(\begin{array}{ll} \mathrm{C} \longleftarrow \mathrm{D} & \\ | & \\ \mathrm{C} & \end{array}\right) + \mathbb{P}\left(\begin{array}{ll} \mathrm{D} \longrightarrow\!\!\!\!\!\!- \mathrm{C} & \\ |\ \swarrow & \\ \mathrm{D} & \end{array}\right) - \mathbb{P}\left(\begin{array}{ll} \mathrm{D} \longleftarrow \mathrm{C} & \\ | & \\ \mathrm{C} & \end{array}\right) - \mathbb{P}\left(\begin{array}{ll} \mathrm{D} \text{ — } \mathrm{D} & \\ |\ \swarrow & \\ \mathrm{C} & \end{array}\right)$$

$$-\sum_{x,y,z,u,v,w}\left[\mathbb{P}\left(\begin{array}{ll} \mathrm{D} & \\ | & \\ \mathrm{C} \longleftarrow \mathrm{D} & \end{array}\right) + \mathbb{P}\left(\begin{array}{ll} \mathrm{D} & \\ |\ \nwarrow\!\!\searrow & \\ \mathrm{C} \text{ — } \mathrm{C} & \end{array}\right)\right].$$

2.2 Self Comparison Spillover

At each discrete time step, a random individual is chosen. With probability p, the chosen individual does inter-layer self comparison spillover updating. Otherwise, with probability $1-p$, she does intra-layer strategy updating. During self comparison spillover updating, the top layer is chosen as the focal layer to **receive** spillover strategies with probability α. Otherwise, with probability $1-\alpha$ the bottom layer will be chosen as the focal layer.

Then, the individual's strategy on the chosen focal layer might be replaced by the strategy of herself on the **opposite** layer. The chance that this might happen depends on payoff comparison, calculated via the Fermi equation, with the exception that the payoff matrix for the iterated prisoners' dilemma game in the bottom layer is normalized by dividing each entry with the parameter m. We denote this normalized payoff matrix by,

$$\begin{pmatrix} \hat{R}_B & \hat{S}_B \\ \hat{T}_B & \hat{P}_B \end{pmatrix}.$$

Let k_T be the average degree of individuals on the top layer. The average payoffs used by self comparison spillover on the top layer are

$$\hat{\pi}_c^T = k_T \cdot q_{c|c} \cdot R_T + k_T \cdot q_{d|c} \cdot S_T,$$

$$\hat{\pi}_d^T = k_T \cdot q_{c|d} \cdot T_T + k_T \cdot q_{d|d} \cdot P_T.$$

And the average normalized payoffs on the bottom layer, used for self comparison spillover updating, are

$$\hat{\pi}_c^B = n(w,x,y,z) \cdot \hat{R}_B + (k_B - n(w,x,y,z)) \cdot \hat{S}_B,$$

$$\hat{\pi}_d^B = n(w,x,y,z) \cdot \hat{T}_B + (k_B - n(w,x,y,z)) \cdot \hat{P}_B,$$

where $n(w,x,y,z)$ is the number of C among neighbours strategies, of the focal individual, on the bottom layer. Then, the probabilities and expected changes in $C-C$ links of events that changes the system through self influence spillover update are as follows.

$$\mathbb{P}\begin{pmatrix} \mathrm{D} \\ \uparrow \\ \mathrm{C} \end{pmatrix} = p \cdot \alpha \cdot F(\hat{\pi}_c^B, \hat{\pi}_d^T) \cdot x_c^d, \qquad \mathbb{P}\begin{pmatrix} \mathrm{D} \\ \downarrow \\ \mathrm{C} \end{pmatrix} = p \cdot (1-\alpha) \cdot F(\hat{\pi}_d^T, \hat{\pi}_c^B) \cdot x_c^d,$$

$$\mathbb{P}\begin{pmatrix} \mathrm{C} \\ \uparrow \\ \mathrm{D} \end{pmatrix} = p \cdot \alpha \cdot F(\hat{\pi}_d^B, \hat{\pi}_c^T) \cdot x_d^c, \qquad \mathbb{P}\begin{pmatrix} \mathrm{C} \\ \downarrow \\ \mathrm{D} \end{pmatrix} = p \cdot (1-\alpha) \cdot F(\hat{\pi}_c^T, \hat{\pi}_d^B) \cdot x_d^c,$$

$$\mathbb{E}\begin{pmatrix} \mathrm{C} \\ \uparrow \\ \mathrm{D} \end{pmatrix} = -k_T \cdot q_{c|c}, \qquad \mathbb{E}\begin{pmatrix} \mathrm{D} \\ \uparrow \\ \mathrm{C} \end{pmatrix} = k_T \cdot q_{c|d},$$

$$\mathbb{E}\begin{pmatrix} \mathrm{D} \\ \downarrow \\ \mathrm{C} \end{pmatrix} = -k_B \cdot p_{c|c}, \qquad \mathbb{E}\begin{pmatrix} \mathrm{C} \\ \downarrow \\ \mathrm{D} \end{pmatrix} = k_B \cdot p_{c|d},$$

where F is the Fermi equation previously defined. Putting it all together, and using the information from the neighbour imitation section, we can write down the differential equations.

$$\dot{q}_c = \mathbb{P}\left(\begin{array}{l} \mathrm{D} \longleftarrow \mathrm{C} \\ \,| \\ \mathrm{C} \end{array}\right) + \mathbb{P}\left(\begin{array}{l} \mathrm{D} \longleftarrow \mathrm{C} \\ \,| \\ \mathrm{D} \end{array}\right) - \mathbb{P}\left(\begin{array}{l} \mathrm{C} \longleftarrow \mathrm{D} \\ \,| \\ \mathrm{C} \end{array}\right) - \mathbb{P}\left(\begin{array}{l} \mathrm{C} \longleftarrow \mathrm{D} \\ \,| \\ \mathrm{D} \end{array}\right)$$

$$+ \sum_{x,y,z,w} \left[\mathbb{P}\begin{pmatrix} \mathrm{D} \\ \uparrow \\ \mathrm{C} \end{pmatrix} - \mathbb{P}\begin{pmatrix} \mathrm{C} \\ \uparrow \\ \mathrm{D} \end{pmatrix} \right].$$

$$\dot{q}_{cc} = \frac{2}{k_T} \cdot \left\{ \mathbb{E}\left(\begin{array}{l} \mathrm{C} \longleftarrow \mathrm{D} \\ \,| \\ \mathrm{C} \end{array}\right) \cdot \mathbb{P}\left(\begin{array}{l} \mathrm{C} \longleftarrow \mathrm{D} \\ \,| \\ \mathrm{C} \end{array}\right) + \mathbb{E}\left(\begin{array}{l} \mathrm{C} \longleftarrow \mathrm{D} \\ \,| \\ \mathrm{D} \end{array}\right) \cdot \mathbb{P}\left(\begin{array}{l} \mathrm{C} \longleftarrow \mathrm{D} \\ \,| \\ \mathrm{D} \end{array}\right) \right.$$

$$+ \mathbb{E}\left(\begin{array}{l} \mathrm{D} \longleftarrow \mathrm{C} \\ \,| \\ \mathrm{C} \end{array}\right) \cdot \mathbb{P}\left(\begin{array}{l} \mathrm{D} \longleftarrow \mathrm{C} \\ \,| \\ \mathrm{C} \end{array}\right) + \mathbb{E}\left(\begin{array}{l} \mathrm{D} \longleftarrow \mathrm{C} \\ \,| \\ \mathrm{D} \end{array}\right) \cdot \mathbb{P}\left(\begin{array}{l} \mathrm{D} \longleftarrow \mathrm{C} \\ \,| \\ \mathrm{D} \end{array}\right)$$

$$\left. + \sum_{x,y,z,w} \left[\mathbb{E}\begin{pmatrix} \mathrm{C} \\ \uparrow \\ \mathrm{D} \end{pmatrix} \cdot \mathbb{P}\begin{pmatrix} \mathrm{C} \\ \uparrow \\ \mathrm{D} \end{pmatrix} + \mathbb{E}\begin{pmatrix} \mathrm{D} \\ \uparrow \\ \mathrm{C} \end{pmatrix} \cdot \mathbb{P}\begin{pmatrix} \mathrm{D} \\ \uparrow \\ \mathrm{C} \end{pmatrix} \right] \right\}.$$

$$\dot{p}_c = \sum_{x,y,z,u,v,w} \left[\mathbb{P}\left(\begin{array}{l} \mathrm{C} \\ \,| \\ \mathrm{D} \longleftarrow \mathrm{C} \end{array}\right) + \mathbb{P}\left(\begin{array}{l} \mathrm{D} \\ \,| \\ \mathrm{D} \longleftarrow \mathrm{C} \end{array}\right) - \mathbb{P}\left(\begin{array}{l} \mathrm{C} \\ \,| \\ \mathrm{C} \longleftarrow \mathrm{D} \end{array}\right) - \mathbb{P}\left(\begin{array}{l} \mathrm{D} \\ \,| \\ \mathrm{C} \longleftarrow \mathrm{D} \end{array}\right) \right]$$

$$+ \sum_{x,y,z,w} \left[\mathbb{P}\begin{pmatrix} \mathrm{C} \\ \downarrow \\ \mathrm{D} \end{pmatrix} - \mathbb{P}\begin{pmatrix} \mathrm{D} \\ \downarrow \\ \mathrm{C} \end{pmatrix} \right].$$

$$\dot{p}_{cc} = \frac{2}{k_T} \cdot \left\{ \sum_{x,y,z,u,v,w} \left[\mathbb{E}\left(\begin{array}{l} \mathrm{D} \\ \,| \\ \mathrm{C} \longleftarrow \mathrm{D} \end{array}\right) \cdot \mathbb{P}\left(\begin{array}{l} \mathrm{D} \\ \,| \\ \mathrm{C} \longleftarrow \mathrm{D} \end{array}\right) + \mathbb{E}\left(\begin{array}{l} \mathrm{C} \\ \,| \\ \mathrm{C} \longleftarrow \mathrm{D} \end{array}\right) \cdot \mathbb{P}\left(\begin{array}{l} \mathrm{C} \\ \,| \\ \mathrm{C} \longleftarrow \mathrm{D} \end{array}\right) \right. \right.$$

$$\left. + \mathbb{E}\left(\begin{array}{l} \mathrm{C} \\ \,| \\ \mathrm{D} \longleftarrow \mathrm{C} \end{array}\right) \cdot \mathbb{P}\left(\begin{array}{l} \mathrm{C} \\ \,| \\ \mathrm{D} \longleftarrow \mathrm{C} \end{array}\right) + \mathbb{E}\left(\begin{array}{l} \mathrm{D} \\ \,| \\ \mathrm{D} \longleftarrow \mathrm{C} \end{array}\right) \cdot \mathbb{P}\left(\begin{array}{l} \mathrm{D} \\ \,| \\ \mathrm{D} \longleftarrow \mathrm{C} \end{array}\right) \right]$$

$$\left. + \sum_{x,y,z,w} \left[\mathbb{E}\begin{pmatrix} \mathrm{C} \\ \downarrow \\ \mathrm{D} \end{pmatrix} \cdot \mathbb{P}\begin{pmatrix} \mathrm{C} \\ \downarrow \\ \mathrm{D} \end{pmatrix} + \mathbb{E}\begin{pmatrix} \mathrm{D} \\ \downarrow \\ \mathrm{C} \end{pmatrix} \cdot \mathbb{P}\begin{pmatrix} \mathrm{D} \\ \downarrow \\ \mathrm{C} \end{pmatrix} \right] \right\}.$$

$$
\begin{aligned}
\dot{x}_c^c = & \sum_{x,y,z,u,v,w} \left[\mathbb{P}\left(\begin{array}{ll} \mathrm{C} & \\ | & \\ \mathrm{D} \longleftarrow & \mathrm{C} \end{array} \right) - \mathbb{P}\left(\begin{array}{ll} \mathrm{C} & \\ | & \\ \mathrm{C} \longleftarrow & \mathrm{D} \end{array} \right) \right] \\
& + \mathbb{P}\left(\begin{array}{ll} \mathrm{D} \longleftarrow & \mathrm{C} \\ | & \\ \mathrm{C} & \end{array} \right) - \mathbb{P}\left(\begin{array}{ll} \mathrm{C} \longleftarrow & \mathrm{D} \\ | & \\ \mathrm{C} & \end{array} \right) \\
& + \sum_{x,y,z,w} \left[\mathbb{P}\left(\begin{array}{c} \mathrm{D} \\ \uparrow \\ \mathrm{C} \end{array} \right) + \mathbb{P}\left(\begin{array}{c} \mathrm{C} \\ \downarrow \\ \mathrm{D} \end{array} \right) \right].
\end{aligned}
$$

$$
\begin{aligned}
\dot{x}_c^d = & \sum_{x,y,z,u,v,w} \left[\mathbb{P}\left(\begin{array}{ll} \mathrm{D} & \\ | & \\ \mathrm{D} \longleftarrow & \mathrm{C} \end{array} \right) - \mathbb{P}\left(\begin{array}{ll} \mathrm{D} & \\ | & \\ \mathrm{C} \longleftarrow & \mathrm{D} \end{array} \right) \right] \\
& + \mathbb{P}\left(\begin{array}{ll} \mathrm{C} \longleftarrow & \mathrm{D} \\ | & \\ \mathrm{C} & \end{array} \right) - \mathbb{P}\left(\begin{array}{ll} \mathrm{D} \longleftarrow & \mathrm{C} \\ | & \\ \mathrm{C} & \end{array} \right) \\
& - \sum_{x,y,z,w} \left[\mathbb{P}\left(\begin{array}{c} \mathrm{D} \\ \uparrow \\ \mathrm{C} \end{array} \right) + \mathbb{P}\left(\begin{array}{c} \mathrm{D} \\ \downarrow \\ \mathrm{C} \end{array} \right) \right].
\end{aligned}
$$

2.3 Context Interference Spillover

At each discrete time step, a random layer and a random individual on that layer is chosen as the focal individual for strategy updating. A random neighbour of this individual is then chosen and their payoffs are compared via the Fermi equation. When calculating payoffs, with probability p, the focal individual experiences context interference and uses her strategy from the bottom layer with probability α and her strategy from the top layer with probability $1-\alpha$. Independently, also with probability p, the chosen neighbour can experience the same context interference. Strategy updating is then carried out with these strategies. The differential equation for the change in fraction of C in the top layer is,

$$
\begin{aligned}
\dot{q}_c = & \mathbb{P}\left(\begin{array}{ll} \mathrm{D} \longleftarrow & \mathrm{C} \\ | & | \\ & \end{array} \right) + \mathbb{P}\left(\begin{array}{ll} \mathrm{D} \leftarrow & \mathrm{D[C]} \\ | & | \\ & \mathrm{C} \end{array} \right) + \mathbb{P}\left(\begin{array}{ll} \mathrm{D[C]} \leftarrow & \mathrm{C} \\ | & | \\ \mathrm{C} & \end{array} \right) + \mathbb{P}\left(\begin{array}{ll} \mathrm{D[C]} \leftarrow & \mathrm{D[C]} \\ | & | \\ \mathrm{C} & \mathrm{C} \end{array} \right) \\
& - \mathbb{P}\left(\begin{array}{ll} \mathrm{C} \longleftarrow & \mathrm{D} \\ | & | \\ & \end{array} \right) - \mathbb{P}\left(\begin{array}{ll} \mathrm{C} \leftarrow & \mathrm{C[D]} \\ | & | \\ & \mathrm{D} \end{array} \right) - \mathbb{P}\left(\begin{array}{ll} \mathrm{C[D]} \leftarrow & \mathrm{D} \\ | & | \\ \mathrm{D} & \end{array} \right) - \mathbb{P}\left(\begin{array}{ll} \mathrm{C[D]} \leftarrow & \mathrm{C[D]} \\ | & | \\ \mathrm{D} & \mathrm{D} \end{array} \right).
\end{aligned}
$$

Detailed equations for each of the terms in the sum are as follows.

$$
\mathbb{P}\left(\begin{array}{ll} \mathrm{D} \longleftarrow & \mathrm{C} \\ | & | \\ & \end{array} \right) = \mathbb{P}\left(\begin{array}{ll} \mathrm{D} \longleftarrow & \mathrm{C} \\ | & | \\ \mathrm{C} & \mathrm{C} \end{array} \right) + \mathbb{P}\left(\begin{array}{ll} \mathrm{D} \longleftarrow & \mathrm{C} \\ | & | \\ \mathrm{C} & \mathrm{D} \end{array} \right) + \mathbb{P}\left(\begin{array}{ll} \mathrm{D} \longleftarrow & \mathrm{C} \\ | & | \\ \mathrm{D} & \mathrm{C} \end{array} \right) + \mathbb{P}\left(\begin{array}{ll} \mathrm{D} \longleftarrow & \mathrm{C} \\ | & | \\ \mathrm{D} & \mathrm{D} \end{array} \right)
$$

$$= \frac{1}{2}\cdot W_{c\to d}\cdot q_d\cdot q_{c|d}\cdot\Bigg(\frac{X_c^d}{q_d}\cdot\frac{X_c^c}{q_c}\cdot(1-p+(1-\alpha)\cdot p)+\frac{X_c^d}{q_d}\cdot\frac{X_d^c}{q_c}\cdot(1-p+(1-\alpha)\cdot p)\cdot(1-p+(1-\alpha)\cdot p)$$
$$+\frac{X_d^d}{q_d}\cdot\frac{X_c^c}{q_c}+\frac{X_d^d}{q_d}\cdot\frac{X_d^c}{q_c}\cdot(1-p+(1-\alpha)\cdot p)\Bigg).$$

$$\mathbb{P}\left(\begin{array}{cc}\mathrm{D}\leftarrow & \mathrm{D[C]}\\ | & | \\ & \mathrm{C}\end{array}\right)=\mathbb{P}\left(\begin{array}{cc}\mathrm{D}\leftarrow & \mathrm{D[C]}\\ | & | \\ \mathrm{C} & \mathrm{C}\end{array}\right)+\mathbb{P}\left(\begin{array}{cc}\mathrm{D}\leftarrow & \mathrm{D[C]}\\ | & | \\ \mathrm{D} & \mathrm{C}\end{array}\right)$$
$$=\frac{1}{2}\cdot W_{c\to d}\cdot q_d\cdot q_{d|d}\cdot\Bigg(\frac{X_c^d}{q_d}\cdot\frac{X_c^d}{q_d}\cdot(1-p+(1-\alpha)\cdot p)\cdot(\alpha\cdot p)+\frac{X_d^d}{q_d}\cdot\frac{X_c^d}{q_c}\cdot(\alpha\cdot p)\Bigg).$$

$$\mathbb{P}\left(\begin{array}{cc}\mathrm{D[C]}\leftarrow & \mathrm{C}\\ | & | \\ \mathrm{C} & \end{array}\right)=\mathbb{P}\left(\begin{array}{cc}\mathrm{D[C]}\leftarrow & \mathrm{C}\\ | & | \\ \mathrm{C} & \mathrm{C}\end{array}\right)+\mathbb{P}\left(\begin{array}{cc}\mathrm{D[C]}\leftarrow & \mathrm{C}\\ | & | \\ \mathrm{C} & \mathrm{D}\end{array}\right)$$
$$=\frac{1}{2}\cdot W_{c\to c}\cdot q_d\cdot q_{c|d}\cdot\frac{X_c^d}{q_d}\cdot(\alpha\cdot p)\cdot\Bigg(\frac{X_c^c}{q_c}+\frac{X_d^c}{q_c}\cdot(1-p+(1-\alpha)\cdot p)\Bigg).$$

$$\mathbb{P}\left(\begin{array}{cc}\mathrm{D[C]}\leftarrow & \mathrm{D[C]}\\ | & | \\ \mathrm{C} & \mathrm{C}\end{array}\right)=\frac{1}{2}\cdot W_{c\to c}\cdot q_d\cdot q_{d|d}\cdot\Bigg(\frac{X_c^d}{q_d}\cdot\frac{X_c^d}{q_d}\cdot(\alpha\cdot p)\cdot(\alpha\cdot p)\Bigg).$$

$$\mathbb{P}\left(\begin{array}{cc}\mathrm{C}\longleftarrow & \mathrm{D}\\ | & | \end{array}\right)=\mathbb{P}\left(\begin{array}{cc}\mathrm{C}\longleftarrow & \mathrm{D}\\ | & | \\ \mathrm{C} & \mathrm{C}\end{array}\right)+\mathbb{P}\left(\begin{array}{cc}\mathrm{C}\longleftarrow & \mathrm{D}\\ | & | \\ \mathrm{C} & \mathrm{D}\end{array}\right)+\mathbb{P}\left(\begin{array}{cc}\mathrm{C}\longleftarrow & \mathrm{D}\\ | & | \\ \mathrm{D} & \mathrm{C}\end{array}\right)+\mathbb{P}\left(\begin{array}{cc}\mathrm{C}\longleftarrow & \mathrm{D}\\ | & | \\ \mathrm{D} & \mathrm{D}\end{array}\right)$$
$$=\frac{1}{2}\cdot W_{d\to c}\cdot q_c\cdot q_{d|c}\cdot\Bigg(\frac{X_c^c}{q_c}\cdot\frac{X_c^d}{q_d}\cdot(1-p+(1-\alpha)\cdot p)+\frac{X_c^c}{q_c}\cdot\frac{X_d^d}{q_d}$$
$$+\frac{X_d^c}{q_c}\cdot\frac{X_c^d}{q_d}\cdot(1-p+(1-\alpha)\cdot p)\cdot(1-p+(1-\alpha)\cdot p)+\frac{X_d^c}{q_c}\cdot\frac{X_d^d}{q_d}\cdot(1-p+(1-\alpha)\cdot p)\Bigg).$$

$$\mathbb{P}\left(\begin{array}{cc}\mathrm{C}\leftarrow & \mathrm{C[D]}\\ | & | \\ & \mathrm{D}\end{array}\right)=\mathbb{P}\left(\begin{array}{cc}\mathrm{C}\leftarrow & \mathrm{C[D]}\\ | & | \\ \mathrm{C} & \mathrm{D}\end{array}\right)+\mathbb{P}\left(\begin{array}{cc}\mathrm{C}\leftarrow & \mathrm{C[D]}\\ | & | \\ \mathrm{D} & \mathrm{D}\end{array}\right)$$
$$=\frac{1}{2}\cdot W_{d\to c}\cdot q_c\cdot q_{c|c}\cdot\frac{X_d^c}{q_c}\cdot(\alpha\cdot p)\cdot\Bigg(\frac{X_c^c}{q_c}+\frac{X_d^c}{q_c}\cdot(1-p+(1-\alpha)\cdot p)\Bigg).$$

$$\mathbb{P}\left(\begin{array}{ccc} \text{C[D]} & \leftarrow & \text{D} \\ | & & | \\ \text{D} & & \end{array}\right) = \mathbb{P}\left(\begin{array}{ccc} \text{C[D]} & \leftarrow & \text{D} \\ | & & | \\ \text{D} & & \text{C} \end{array}\right) + \mathbb{P}\left(\begin{array}{ccc} \text{C[D]} & \leftarrow & \text{D} \\ | & & | \\ \text{D} & & \text{D} \end{array}\right)$$

$$= \frac{1}{2} \cdot W_{d\to d} \cdot q_c \cdot q_{d|c} \cdot \frac{X_d^c}{q_c} \cdot (\alpha \cdot p) \cdot \left(\frac{X_c^d}{q_d} \cdot (1 - p + (1-\alpha) \cdot p) + \frac{X_d^d}{q_d} \right).$$

$$\mathbb{P}\left(\begin{array}{ccc} \text{C[D]} & \longleftarrow & \text{C[D]} \\ | & & | \\ \text{D} & & \text{D} \end{array}\right) = \frac{1}{2} \cdot W_{d\to d} \cdot q_c \cdot q_{c|c} \cdot \left(\frac{X_d^c}{q_c} \cdot \frac{X_d^c}{q_c} \cdot (\alpha \cdot p) \cdot (\alpha \cdot p) \right)$$

We write the differential equation for the change in fraction of $C - C$ links on the top layer as a sum,

$$\dot{q}_{cc} = \frac{2}{k_T} \sum_E \mathbb{P}(E)\mathbb{E}(E),$$

where the summation is over all events E that results in a change in q_c, and k_T is the average degree of individual in the top network layer. All of the $\mathbb{P}(E)$ have been stated above. The values of $\mathbb{E}(E)$, the expected change in fraction of $C - C$ links are as follows.

$$\mathbb{E}\left(\begin{array}{ccc} \text{D} & \longleftarrow & \text{C} \\ | & & | \end{array}\right) = (k_T - 1) \cdot q_{c|d} + 1, \qquad \mathbb{E}\left(\begin{array}{ccc} \text{D} & \longleftarrow & \text{D[C]} \\ | & & | \end{array}\right) = (k_T - 1) \cdot q_{c|d}.$$

$$\mathbb{E}\left(\begin{array}{ccc} \text{D[C]} & \leftarrow & \text{C} \\ | & & | \\ \text{C} & & \end{array}\right) = (k_T - 1) \cdot q_{c|d} + 1, \qquad \mathbb{E}\left(\begin{array}{ccc} \text{D[C]} & \leftarrow & \text{D[C]} \\ | & & | \\ \text{C} & & \text{C} \end{array}\right) = (k_T - 1) \cdot q_{c|d}.$$

$$\mathbb{E}\left(\begin{array}{ccc} \text{C} & \longleftarrow & \text{D} \\ | & & | \end{array}\right) = -(k_T - 1) \cdot q_{c|c}, \qquad \mathbb{E}\left(\begin{array}{ccc} \text{C} & \longleftarrow & \text{C[D]} \\ | & & | \end{array}\right) = -(k_T - 1) \cdot q_{c|c} - 1.$$

$$\mathbb{E}\left(\begin{array}{ccc} \text{C[D]} & \leftarrow & \text{D} \\ | & & | \\ \text{D} & & \end{array}\right) = -(k_T - 1) \cdot q_{c|c}, \qquad \mathbb{E}\left(\begin{array}{ccc} \text{C[D]} & \leftarrow & \text{C[D]} \\ | & & | \\ \text{D} & & \text{D} \end{array}\right) = -(k_T - 1) \cdot q_{c|c} - 1.$$

The differential equation for the change in fraction of C in the bottom layer is,

$$\dot{p}_c = \sum_{x,y,z,u,v,w} \Bigg[\mathbb{P}\left(\begin{array}{ccc} | & & | \\ \text{D} & \longleftarrow & \text{C} \end{array}\right) + \mathbb{P}\left(\begin{array}{ccc} & & \text{C} \\ | & & | \\ \text{D} & \leftarrow & \text{D[C]} \end{array}\right) + \mathbb{P}\left(\begin{array}{ccc} \text{C} & & \\ | & & | \\ \text{D[C]} & \leftarrow & \text{C} \end{array}\right) + \mathbb{P}\left(\begin{array}{ccc} \text{C} & & \text{C} \\ | & & | \\ \text{D[C]} & \longleftarrow & \text{D[C]} \end{array}\right)$$

$$- \mathbb{P}\left(\begin{array}{ccc} | & & | \\ \text{C} & \longleftarrow & \text{D} \end{array}\right) - \mathbb{P}\left(\begin{array}{ccc} & & \text{D} \\ | & & | \\ \text{C} & \leftarrow & \text{C[D]} \end{array}\right) - \mathbb{P}\left(\begin{array}{ccc} \text{D} & & \\ | & & | \\ \text{C[D]} & \leftarrow & \text{D} \end{array}\right) - \mathbb{P}\left(\begin{array}{ccc} \text{D} & & \text{D} \\ | & & | \\ \text{C[D]} & \longleftarrow & \text{C[D]} \end{array}\right) \Bigg],$$

where the summation is over all possible strategies of the neighbours x, y, z of the focal individual, and over all possible strategies u, v, w of the neighbours of the non-focal individual. Detailed equations for each of the terms in the sum are as follows.

$$\mathbb{P}\left(\begin{array}{ccc} & & \\ | & & | \\ \mathrm{D} & \longleftarrow & \mathrm{C} \end{array}\right) = \mathbb{P}\left(\begin{array}{ccc} \mathrm{C} & & \mathrm{C} \\ | & & | \\ \mathrm{D} & \longleftarrow & \mathrm{C} \end{array}\right) + \mathbb{P}\left(\begin{array}{ccc} \mathrm{C} & & \mathrm{D} \\ | & & | \\ \mathrm{D} & \longleftarrow & \mathrm{C} \end{array}\right) + \mathbb{P}\left(\begin{array}{ccc} \mathrm{D} & & \mathrm{C} \\ | & & | \\ \mathrm{D} & \longleftarrow & \mathrm{C} \end{array}\right) + \mathbb{P}\left(\begin{array}{ccc} \mathrm{D} & & \mathrm{D} \\ | & & | \\ \mathrm{D} & \longleftarrow & \mathrm{C} \end{array}\right)$$

$$= \frac{1}{2} \cdot \phi_{c \to d} \cdot p_d \cdot p_{c|d} \cdot \left(\frac{X_d^c}{p_d} \cdot \frac{X_c^c}{p_c} \cdot (1 - p + \alpha \cdot p) + \frac{X_d^c}{p_d} \cdot \frac{X_c^d}{p_c} \cdot (1 - p + \alpha \cdot p) \cdot (1 - p + \alpha \cdot p) \right.$$

$$\left. + \frac{X_d^d}{p_d} \cdot \frac{X_c^c}{p_c} + \frac{X_d^d}{p_d} \cdot \frac{X_c^d}{p_c} \cdot (1 - p + \alpha \cdot p) \right).$$

$$\mathbb{P}\left(\begin{array}{ccc} & & \mathrm{C} \\ | & & | \\ \mathrm{D} & \leftarrow & \mathrm{D[C]} \end{array}\right) = \mathbb{P}\left(\begin{array}{ccc} \mathrm{C} & & \mathrm{C} \\ | & & | \\ \mathrm{D} & \leftarrow & \mathrm{D[C]} \end{array}\right) + \mathbb{P}\left(\begin{array}{ccc} \mathrm{D} & & \mathrm{C} \\ | & & | \\ \mathrm{D} & \leftarrow & \mathrm{D[C]} \end{array}\right)$$

$$= \frac{1}{2} \cdot \phi_{c \to d} \cdot p_d \cdot p_{d|d} \cdot \frac{X_d^c}{p_d} \cdot ((1 - \alpha) \cdot p) \cdot \left(\frac{X_d^c}{p_d} \cdot (1 - p + \alpha \cdot p) + \frac{X_d^d}{p_d} \right).$$

$$\mathbb{P}\left(\begin{array}{ccc} \mathrm{C} & & \\ | & & | \\ \mathrm{D[C]} & \leftarrow & \mathrm{C} \end{array}\right) = \mathbb{P}\left(\begin{array}{ccc} \mathrm{C} & & \mathrm{D} \\ | & & | \\ \mathrm{D[C]} & \leftarrow & \mathrm{C} \end{array}\right) + \mathbb{P}\left(\begin{array}{ccc} \mathrm{C} & & \mathrm{C} \\ | & & | \\ \mathrm{D[C]} & \leftarrow & \mathrm{C} \end{array}\right)$$

$$= \frac{1}{2} \cdot \phi_{c \to c} \cdot p_d \cdot p_{c|d} \cdot \frac{X_d^c}{p_d} \cdot ((1 - \alpha) \cdot p) \cdot \left(\frac{X_c^d}{p_c} \cdot (1 - p + \alpha \cdot p) + \frac{X_c^c}{p_c} \right).$$

$$\mathbb{P}\left(\begin{array}{ccc} \mathrm{C} & & \mathrm{C} \\ | & & | \\ \mathrm{D[C]} & \longleftarrow & \mathrm{D[C]} \end{array}\right) = \frac{1}{2} \cdot \phi_{c \to c} \cdot p_d \cdot p_{d|d} \cdot \left(\frac{X_d^c}{p_d} \cdot \frac{X_d^c}{p_d} \cdot ((1 - \alpha) \cdot p) \cdot ((1 - \alpha) \cdot p) \right).$$

$$\mathbb{P}\left(\begin{array}{ccc} & & \\ | & & | \\ \mathrm{C} & \longleftarrow & \mathrm{D} \end{array}\right) = \mathbb{P}\left(\begin{array}{ccc} \mathrm{C} & & \mathrm{C} \\ | & & | \\ \mathrm{C} & \longleftarrow & \mathrm{D} \end{array}\right) + \mathbb{P}\left(\begin{array}{ccc} \mathrm{C} & & \mathrm{D} \\ | & & | \\ \mathrm{C} & \longleftarrow & \mathrm{D} \end{array}\right) + \mathbb{P}\left(\begin{array}{ccc} \mathrm{D} & & \mathrm{C} \\ | & & | \\ \mathrm{C} & \longleftarrow & \mathrm{D} \end{array}\right) + \mathbb{P}\left(\begin{array}{ccc} \mathrm{D} & & \mathrm{D} \\ | & & | \\ \mathrm{C} & \longleftarrow & \mathrm{D} \end{array}\right)$$

$$= \frac{1}{2} \cdot \phi_{d \to c} \cdot p_c \cdot p_{d|c} \cdot \left(\frac{X_c^c}{p_c} \cdot \frac{X_d^c}{p_d} \cdot (1 - p + \alpha \cdot p) + \frac{X_c^c}{p_c} \cdot \frac{X_d^d}{p_d} \right.$$

$$\left. + \frac{X_c^d}{p_c} \cdot \frac{X_d^c}{p_d} \cdot (1 - p + \alpha \cdot p) \cdot (1 - p + \alpha \cdot p) + \frac{X_c^d}{p_c} \cdot \frac{X_d^d}{p_d} \cdot (1 - p + \alpha \cdot p) \right).$$

$$\mathbb{P}\left(\begin{array}{ccc} & & \mathrm{D} \\ | & & | \\ \mathrm{C} & \leftarrow & \mathrm{C[D]} \end{array}\right) = \mathbb{P}\left(\begin{array}{ccc} \mathrm{D} & & \mathrm{D} \\ | & & | \\ \mathrm{C} & \leftarrow & \mathrm{C[D]} \end{array}\right) + \mathbb{P}\left(\begin{array}{ccc} \mathrm{C} & & \mathrm{D} \\ | & & | \\ \mathrm{C} & \leftarrow & \mathrm{C[D]} \end{array}\right)$$

$$= \frac{1}{2} \cdot \phi_{d \to c} \cdot p_c \cdot p_{c|c} \cdot \frac{X_c^d}{p_c} \cdot ((1-\alpha) \cdot p) \cdot \left(\frac{X_c^d}{p_c} \cdot (1 - p + \alpha \cdot p) + \frac{X_c^c}{p_c} \right).$$

$$\mathbb{P}\left(\begin{array}{ccc} \mathrm{D} & & \\ | & & | \\ \mathrm{C[D]} & \leftarrow & \mathrm{D} \end{array} \right) = \mathbb{P}\left(\begin{array}{ccc} \mathrm{D} & & \mathrm{C} \\ | & & | \\ \mathrm{C[D]} & \leftarrow & \mathrm{D} \end{array} \right) + \mathbb{P}\left(\begin{array}{ccc} \mathrm{D} & & \mathrm{D} \\ | & & | \\ \mathrm{C[D]} & \leftarrow & \mathrm{D} \end{array} \right)$$

$$= \frac{1}{2} \cdot \phi_{d \to d} \cdot p_c \cdot p_{d|c} \cdot \frac{X_c^d}{p_c} \cdot ((1-\alpha) \cdot p) \cdot \left(\frac{X_d^c}{p_d} \cdot (1 - p + \alpha \cdot p) + \frac{X_d^d}{p_d} \right).$$

$$\mathbb{P}\left(\begin{array}{ccc} \mathrm{D} & & \mathrm{D} \\ | & & | \\ \mathrm{C[D]} & \longleftarrow & \mathrm{C[D]} \end{array} \right) = \frac{1}{2} \cdot \phi_{d \to d} \cdot p_c \cdot p_{c|c} \cdot \left(\frac{X_c^d}{p_c} \cdot \frac{X_c^d}{p_c} \cdot ((1-\alpha) \cdot p) \cdot ((1-\alpha) \cdot p) \right).$$

We write the differential equation for the change in fraction of $C-C$ links on the bottom layer as a sum,

$$\dot{p}_{cc} = \frac{2}{k_B} \sum_{E} \sum_{x,y,z,u,v,w} \mathbb{P}(E)\mathbb{E}(E),$$

where the summation is over all possible strategies of the neighbours x, y, z of the focal individual, over all possible strategies u, v, w of the neighbours of the non-focal individual, and all events E that results in a change in p_c. k_B is the average degree of the bottom layer. All of the $\mathbb{P}(E)$ have been defined above. Let $n(x, y, z)$ be the number of C among neighbour strategies x, y, z. Then $\mathbb{E}(E)$, the expected change in fraction of $C-C$ links for each event E are as follows.

$$\mathbb{E}\left(\begin{array}{ccc} | & & | \\ \mathrm{D} & \longleftarrow & \mathrm{C} \end{array} \right) = n(x,y,z) + 1, \qquad \mathbb{E}\left(\begin{array}{ccc} | & & | \\ \mathrm{D} & \leftarrow & \mathrm{D[C]} \end{array} \right) = n(x,y,z).$$

$$\mathbb{E}\left(\begin{array}{ccc} \mathrm{C} & & \\ | & & | \\ \mathrm{D[C]} & \leftarrow & \mathrm{C} \end{array} \right) = n(x,y,z) + 1, \qquad \mathbb{E}\left(\begin{array}{ccc} \mathrm{C} & & \mathrm{C} \\ | & & | \\ \mathrm{D[C]} & \longleftarrow & \mathrm{D[C]} \end{array} \right) = n(x,y,z).$$

$$\mathbb{E}\left(\begin{array}{ccc} | & & | \\ \mathrm{C} & \longleftarrow & \mathrm{D} \end{array} \right) = -n(x,y,z), \qquad \mathbb{E}\left(\begin{array}{ccc} | & & | \\ \mathrm{C} & \leftarrow & \mathrm{C[D]} \end{array} \right) = -n(x,y,z) - 1.$$

$$\mathbb{E}\left(\begin{array}{ccc} \mathrm{D} & & \\ | & & | \\ \mathrm{C[D]} & \leftarrow & \mathrm{D} \end{array} \right) = -n(x,y,z), \qquad \mathbb{E}\left(\begin{array}{ccc} \mathrm{D} & & \mathrm{D} \\ | & & | \\ \mathrm{C[D]} & \longleftarrow & \mathrm{C[D]} \end{array} \right) = -n(x,y,z) - 1.$$

The differential equation for the change in fraction of individuals with strategy C on the top layer and C in the bottom layer is,

$$\dot{X}_c^c = -\mathbb{P}\begin{pmatrix} \mathrm{C} & \longleftarrow & \mathrm{D} \\ | & & | \\ \mathrm{C} & & \end{pmatrix} - \mathbb{P}\begin{pmatrix} \mathrm{C} & \leftarrow & \mathrm{C[D]} \\ | & & | \\ \mathrm{C} & & \mathrm{D} \end{pmatrix} - \sum_{x,y,z,u,v,w} \mathbb{P}\begin{pmatrix} \mathrm{C} & & \\ | & & | \\ \mathrm{C} & \longleftarrow & \mathrm{D} \end{pmatrix} - \sum_{x,y,z,u,v,w} \mathbb{P}\begin{pmatrix} \mathrm{C} & & \mathrm{D} \\ | & & | \\ \mathrm{C} & \leftarrow & \mathrm{C[D]} \end{pmatrix}$$

$$+\mathbb{P}\begin{pmatrix} \mathrm{D} & \longleftarrow & \mathrm{C} \\ | & & | \\ \mathrm{C} & & \end{pmatrix} + \mathbb{P}\begin{pmatrix} \mathrm{D} & \leftarrow & \mathrm{D[C]} \\ | & & | \\ \mathrm{C} & & \mathrm{C} \end{pmatrix} + \mathbb{P}\begin{pmatrix} \mathrm{D[C]} & \leftarrow & \mathrm{D[C]} \\ | & & | \\ \mathrm{C} & & \mathrm{C} \end{pmatrix} + \mathbb{P}\begin{pmatrix} \mathrm{D[C]} & \leftarrow & \mathrm{C} \\ | & & | \\ \mathrm{C} & & \end{pmatrix}$$

$$+ \sum_{x,y,z,u,v,w} \mathbb{P}\begin{pmatrix} \mathrm{C} & & \\ | & & | \\ \mathrm{D} & \longleftarrow & \mathrm{C} \end{pmatrix} + \sum_{x,y,z,u,v,w} \mathbb{P}\begin{pmatrix} \mathrm{C} & & \mathrm{C} \\ | & & | \\ \mathrm{D} & \leftarrow & \mathrm{D[C]} \end{pmatrix} + \sum_{x,y,z,u,v,w} \mathbb{P}\begin{pmatrix} \mathrm{C} & & \mathrm{C} \\ | & & | \\ \mathrm{D[C]} & \leftarrow & \mathrm{D[C]} \end{pmatrix} + \sum_{x,y,z,u,v,w} \mathbb{P}\begin{pmatrix} \mathrm{C} & & \\ | & & | \\ \mathrm{D[C]} & \longleftarrow & \mathrm{C} \end{pmatrix}.$$

Details for each term in the sum are as follows.

$$\mathbb{P}\begin{pmatrix} \mathrm{C} & \longleftarrow & \mathrm{D} \\ | & & | \\ \mathrm{C} & & \end{pmatrix} = \mathbb{P}\begin{pmatrix} \mathrm{C} & \longleftarrow & \mathrm{D} \\ | & & | \\ \mathrm{C} & & \mathrm{D} \end{pmatrix} + \mathbb{P}\begin{pmatrix} \mathrm{C} & \longleftarrow & \mathrm{D} \\ | & & | \\ \mathrm{C} & & \mathrm{C} \end{pmatrix}$$

$$= \frac{1}{2} \cdot W_{d\to c} \cdot q_c \cdot q_{d|c} \cdot \frac{X_c^c}{q_c} \cdot \left(\frac{X_d^d}{q_d} + \frac{X_c^d}{q_d} \cdot (1 - p + (1-\alpha) \cdot p) \right).$$

$$\mathbb{P}\begin{pmatrix} \mathrm{C} & \leftarrow & \mathrm{C[D]} \\ | & & | \\ \mathrm{C} & & \mathrm{D} \end{pmatrix} = \frac{1}{2} \cdot W_{d\to c} \cdot q_c \cdot q_{c|c} \cdot \frac{X_c^c}{q_c} \cdot \frac{X_d^c}{q_c} \cdot (\alpha \cdot p).$$

$$\mathbb{P}\begin{pmatrix} \mathrm{C} & & \\ | & & | \\ \mathrm{C} & \longleftarrow & \mathrm{D} \end{pmatrix} = \mathbb{P}\begin{pmatrix} \mathrm{C} & & \mathrm{C} \\ | & & | \\ \mathrm{C} & \longleftarrow & \mathrm{D} \end{pmatrix} + \mathbb{P}\begin{pmatrix} \mathrm{C} & & \mathrm{D} \\ | & & | \\ \mathrm{C} & \longleftarrow & \mathrm{D} \end{pmatrix}$$

$$= \frac{1}{2} \cdot \phi_{d\to c} \cdot p_c \cdot p_{d|c} \cdot \frac{X_c^c}{p_c} \cdot \left(\frac{X_d^c}{p_d} \cdot (1 - p + \alpha \cdot p) + \frac{X_d^d}{p_d} \right).$$

$$\mathbb{P}\begin{pmatrix} \mathrm{C} & & \mathrm{D} \\ | & & | \\ \mathrm{C} & \leftarrow & \mathrm{C[D]} \end{pmatrix} = \frac{1}{2} \cdot \phi_{d\to c} \cdot p_c \cdot p_{c|c} \cdot \frac{X_c^c}{p_c} \cdot \frac{X_c^d}{p_c} \cdot ((1-\alpha) \cdot p).$$

$$\mathbb{P}\begin{pmatrix} \mathrm{D} & \longleftarrow & \mathrm{C} \\ | & & | \\ \mathrm{C} & & \end{pmatrix} = \mathbb{P}\begin{pmatrix} \mathrm{D} & \longleftarrow & \mathrm{C} \\ | & & | \\ \mathrm{C} & & \mathrm{D} \end{pmatrix} + \mathbb{P}\begin{pmatrix} \mathrm{D} & \longleftarrow & \mathrm{C} \\ | & & | \\ \mathrm{C} & & \mathrm{C} \end{pmatrix}$$

$$= \frac{1}{2} \cdot W_{c \to d} \cdot q_d \cdot q_{c|d} \cdot \frac{X_c^d}{q_d} \cdot (1 - p + (1-\alpha) \cdot p) \cdot \left(\frac{X_d^c}{q_c} \cdot (1 - p + (1-\alpha) \cdot p) + \frac{X_c^c}{q_c} \right).$$

$$\mathbb{P}\left(\begin{array}{ccc} \text{D} & \leftarrow & \text{D[C]} \\ | & & | \\ \text{C} & & \text{C} \end{array} \right) = \frac{1}{2} \cdot W_{c \to d} \cdot q_d \cdot q_{d|d} \cdot \frac{X_c^d}{q_d} \cdot (1 - p + (1-\alpha) \cdot p) \cdot \frac{X_c^d}{q_d} \cdot (\alpha \cdot p).$$

$$\mathbb{P}\left(\begin{array}{ccc} \text{D[C]} & \longleftarrow & \text{D[C]} \\ | & & | \\ \text{C} & & \text{C} \end{array} \right) = \frac{1}{2} \cdot W_{c \to c} \cdot q_d \cdot q_{d|d} \cdot \frac{X_c^d}{q_d} \cdot (\alpha \cdot p) \cdot \frac{X_c^d}{q_d} \cdot (\alpha \cdot p).$$

$$\mathbb{P}\left(\begin{array}{ccc} \text{D[C]} & \leftarrow & \text{C} \\ | & & | \\ \text{C} & & \end{array} \right) = \mathbb{P}\left(\begin{array}{ccc} \text{D[C]} & \leftarrow & \text{C} \\ | & & | \\ \text{C} & & \text{D} \end{array} \right) + \mathbb{P}\left(\begin{array}{ccc} \text{D[C]} & \leftarrow & \text{C} \\ | & & | \\ \text{C} & & \text{C} \end{array} \right)$$

$$= \frac{1}{2} \cdot W_{c \to c} \cdot q_d \cdot q_{c|d} \cdot \frac{X_c^d}{q_d} \cdot (\alpha \cdot p) \cdot \left(\frac{X_d^c}{q_c} \cdot (1 - p + (1-\alpha) \cdot p) + \frac{X_c^c}{q_c} \right).$$

$$\mathbb{P}\left(\begin{array}{ccc} \text{C} & & \\ | & & | \\ \text{D} & \longleftarrow & \text{C} \end{array} \right) = \mathbb{P}\left(\begin{array}{ccc} \text{C} & & \text{C} \\ | & & | \\ \text{D} & \longleftarrow & \text{C} \end{array} \right) + \mathbb{P}\left(\begin{array}{ccc} \text{C} & & \text{D} \\ | & & | \\ \text{D} & \longleftarrow & \text{C} \end{array} \right)$$

$$= \frac{1}{2} \cdot \phi_{c \to d} \cdot p_d \cdot p_{c|d} \cdot \frac{X_d^c}{p_d} \cdot (1 - p + \alpha \cdot p) \cdot \left(\frac{X_c^c}{p_c} + \frac{X_c^d}{p_c} \cdot (1 - p + \alpha \cdot p) \right).$$

$$\mathbb{P}\left(\begin{array}{ccc} \text{C} & & \text{C} \\ | & & | \\ \text{D} & \leftarrow & \text{D[C]} \end{array} \right) = \frac{1}{2} \cdot \phi_{c \to d} \cdot p_d \cdot p_{d|d} \cdot \frac{X_d^c}{p_d} \cdot (1 - p + \alpha \cdot p) \cdot \frac{X_d^c}{p_d} \cdot ((1-\alpha) \cdot p).$$

$$\mathbb{P}\left(\begin{array}{ccc} \text{C} & & \text{C} \\ | & & | \\ \text{D[C]} & \longleftarrow & \text{D[C]} \end{array} \right) = \frac{1}{2} \cdot \phi_{c \to c} \cdot p_d \cdot p_{d|d} \cdot \frac{X_d^c}{p_d} \cdot ((1-\alpha) \cdot p) \cdot \frac{X_d^c}{p_d} \cdot ((1-\alpha) \cdot p).$$

$$\mathbb{P}\left(\begin{array}{ccc} \text{C} & & \\ | & & | \\ \text{D[C]} & \leftarrow & \text{C} \end{array} \right) = \mathbb{P}\left(\begin{array}{ccc} \text{C} & & \text{D} \\ | & & | \\ \text{D[C]} & \leftarrow & \text{C} \end{array} \right) + \mathbb{P}\left(\begin{array}{ccc} \text{C} & & \text{C} \\ | & & | \\ \text{D[C]} & \leftarrow & \text{C} \end{array} \right)$$

$$= \frac{1}{2} \cdot \phi_{c \to c} \cdot p_d \cdot p_{c|d} \cdot \frac{X_d^c}{p_d} \cdot ((1-\alpha) \cdot p) \cdot \left(\frac{X_c^d}{p_c} \cdot (1 - p + \alpha \cdot p) + \frac{X_c^c}{p_c} \right).$$

The differential equation for the change in fraction of individuals with strategy D on the top layer and C in the bottom layer is,

$$\dot{X}_c^d = -\mathbb{P}\begin{pmatrix} \text{D} & \longleftarrow & \text{C} \\ | & & | \\ \text{C} & & \end{pmatrix} - \mathbb{P}\begin{pmatrix} \text{D} & \leftarrow & \text{D[C]} \\ | & & | \\ \text{C} & & \text{C} \end{pmatrix} - \sum_{x,y,z,u,v,w} \mathbb{P}\begin{pmatrix} \text{D} & & \\ | & & | \\ \text{C} & \longleftarrow & \text{D} \end{pmatrix} - \sum_{x,y,z,u,v,w} \mathbb{P}\begin{pmatrix} \text{D} & & \text{D} \\ | & & | \\ \text{C} & \leftarrow & \text{C[D]} \end{pmatrix}$$

$$-\mathbb{P}\begin{pmatrix} \text{D[C]} & \leftarrow & \text{C} \\ | & & | \\ \text{C} & & \end{pmatrix} - \mathbb{P}\begin{pmatrix} \text{D[C]} & \longleftarrow & \text{D[C]} \\ | & & | \\ \text{C} & & \text{C} \end{pmatrix} - \sum_{x,y,z,u,v,w} \mathbb{P}\begin{pmatrix} \text{D} & & \\ | & & | \\ \text{C[D]} & \leftarrow & \text{D} \end{pmatrix} - \sum_{x,y,z,u,v,w} \mathbb{P}\begin{pmatrix} \text{D} & & \text{D} \\ | & & | \\ \text{C[D]} & \longleftarrow & \text{C[D]} \end{pmatrix}$$

$$+\mathbb{P}\begin{pmatrix} \text{C} & \longleftarrow & \text{D} \\ | & & | \\ \text{C} & & \end{pmatrix} + \mathbb{P}\begin{pmatrix} \text{C} & \leftarrow & \text{C[D]} \\ | & & | \\ \text{C} & & \text{D} \end{pmatrix} + \sum_{x,y,z,u,v,w} \mathbb{P}\begin{pmatrix} \text{D} & & \\ | & & | \\ \text{D} & \longleftarrow & \text{C} \end{pmatrix} + \sum_{x,y,z,u,v,w} \mathbb{P}\begin{pmatrix} \text{D} & & \text{C} \\ | & & | \\ \text{D} & \leftarrow & \text{D[C]} \end{pmatrix}.$$

Details for each term in the sum are as follows.

$$\mathbb{P}\begin{pmatrix} \text{D} & \longleftarrow & \text{C} \\ | & & | \\ \text{C} & & \end{pmatrix} = \mathbb{P}\begin{pmatrix} \text{D} & \longleftarrow & \text{C} \\ | & & | \\ \text{C} & & \text{C} \end{pmatrix} + \mathbb{P}\begin{pmatrix} \text{D} & \longleftarrow & \text{C} \\ | & & | \\ \text{C} & & \text{D} \end{pmatrix}$$

$$= \frac{1}{2} \cdot W_{c \to d} \cdot q_d \cdot q_{c|d} \cdot \frac{X_c^d}{q_d} \cdot (1 - p + (1-\alpha) \cdot p) \cdot \left(\frac{X_c^c}{q_c} + \frac{X_d^c}{q_c} \cdot (1 - p + (1-\alpha) \cdot p) \right).$$

$$\mathbb{P}\begin{pmatrix} \text{D} & \leftarrow & \text{D[C]} \\ | & & | \\ \text{C} & & \text{C} \end{pmatrix} = \frac{1}{2} \cdot W_{c \to d} \cdot q_d \cdot q_{d|d} \cdot \frac{X_c^d}{q_d} \cdot (1 - p + (1-\alpha) \cdot p) \cdot \frac{X_c^d}{q_d} \cdot (\alpha \cdot p).$$

$$\mathbb{P}\begin{pmatrix} \text{D[C]} & \leftarrow & \text{C} \\ | & & | \\ \text{C} & & \end{pmatrix} = \mathbb{P}\begin{pmatrix} \text{D[C]} & \leftarrow & \text{C} \\ | & & | \\ \text{C} & & \text{C} \end{pmatrix} + \mathbb{P}\begin{pmatrix} \text{D[C]} & \leftarrow & \text{C} \\ | & & | \\ \text{C} & & \text{D} \end{pmatrix}$$

$$= \frac{1}{2} \cdot W_{c \to c} \cdot q_d \cdot q_{c|d} \cdot \frac{X_c^d}{q_d} \cdot (\alpha \cdot p) \cdot \left(\frac{X_c^c}{q_c} + \frac{X_d^c}{q_c} \cdot (1 - p + (1-\alpha) \cdot p) \right).$$

$$\mathbb{P}\begin{pmatrix} \text{D[C]} & \longleftarrow & \text{D[C]} \\ | & & | \\ \text{C} & & \text{C} \end{pmatrix} = \frac{1}{2} \cdot W_{c \to c} \cdot q_d \cdot q_{d|d} \cdot \frac{X_c^d}{q_d} \cdot (\alpha \cdot p) \cdot \frac{X_c^d}{q_d} \cdot (\alpha \cdot p).$$

$$\mathbb{P}\left(\begin{array}{ccc} \mathrm{D} & & \\ | & & | \\ \mathrm{C} & \longleftarrow & \mathrm{D} \end{array}\right) = \mathbb{P}\left(\begin{array}{ccc} \mathrm{D} & & \mathrm{C} \\ | & & | \\ \mathrm{C} & \longleftarrow & \mathrm{D} \end{array}\right) + \mathbb{P}\left(\begin{array}{ccc} \mathrm{D} & & \mathrm{D} \\ | & & | \\ \mathrm{C} & \longleftarrow & \mathrm{D} \end{array}\right)$$

$$= \frac{1}{2} \cdot \phi_{d\to c} \cdot p_c \cdot p_{d|c} \cdot \frac{X_c^d}{p_c} \cdot (1 - p + \alpha \cdot p) \cdot \left(\frac{X_d^c}{p_d} \cdot (1 - p + \alpha \cdot p) + \frac{X_d^d}{p_d}\right).$$

$$\mathbb{P}\left(\begin{array}{ccc} \mathrm{D} & & \mathrm{D} \\ | & & | \\ \mathrm{C} & \leftarrow & \mathrm{C[D]} \end{array}\right) = \frac{1}{2} \cdot \phi_{d\to c} \cdot p_c \cdot p_{c|c} \cdot \frac{X_c^d}{p_c} \cdot (1 - p + \alpha \cdot p) \cdot \frac{X_c^d}{p_c} \cdot ((1 - \alpha) \cdot p).$$

$$\mathbb{P}\left(\begin{array}{ccc} \mathrm{D} & & \\ | & & | \\ \mathrm{C[D]} & \leftarrow & \mathrm{D} \end{array}\right) = \mathbb{P}\left(\begin{array}{ccc} \mathrm{D} & & \mathrm{C} \\ | & & | \\ \mathrm{C[D]} & \leftarrow & \mathrm{D} \end{array}\right) + \mathbb{P}\left(\begin{array}{ccc} \mathrm{D} & & \mathrm{D} \\ | & & | \\ \mathrm{C[D]} & \leftarrow & \mathrm{D} \end{array}\right)$$

$$= \frac{1}{2} \cdot \phi_{d\to d} \cdot p_c \cdot p_{d|c} \cdot \frac{X_c^d}{p_c} \cdot ((1 - \alpha) \cdot p) \cdot \left(\frac{X_d^c}{p_d} \cdot (1 - p + \alpha \cdot p) + \frac{X_d^d}{p_d}\right).$$

$$\mathbb{P}\left(\begin{array}{ccc} \mathrm{D} & & \mathrm{D} \\ | & & | \\ \mathrm{C[D]} & \longleftarrow & \mathrm{C[D]} \end{array}\right) = \frac{1}{2} \cdot \phi_{d\to d} \cdot p_c \cdot p_{c|c} \cdot \frac{X_c^d}{p_c} \cdot ((1 - \alpha) \cdot p) \cdot \frac{X_c^d}{p_c} \cdot ((1 - \alpha) \cdot p).$$

$$\mathbb{P}\left(\begin{array}{ccc} \mathrm{C} & \longleftarrow & \mathrm{D} \\ | & & | \\ \mathrm{C} & & \end{array}\right) = \mathbb{P}\left(\begin{array}{ccc} \mathrm{C} & \longleftarrow & \mathrm{D} \\ | & & | \\ \mathrm{C} & & \mathrm{C} \end{array}\right) + \mathbb{P}\left(\begin{array}{ccc} \mathrm{C} & \longleftarrow & \mathrm{D} \\ | & & | \\ \mathrm{C} & & \mathrm{D} \end{array}\right)$$

$$= \frac{1}{2} \cdot W_{d\to c} \cdot q_c \cdot q_{d|c} \cdot \frac{X_c^c}{q_c} \cdot \left(\frac{X_c^d}{q_d} \cdot (1 - p + (1 - \alpha) \cdot p) + \frac{X_d^d}{q_d}\right).$$

$$\mathbb{P}\left(\begin{array}{ccc} \mathrm{C} & \leftarrow & \mathrm{C[D]} \\ | & & | \\ \mathrm{C} & & \mathrm{D} \end{array}\right) = \frac{1}{2} \cdot W_{d\to c} \cdot q_c \cdot q_{c|c} \cdot \frac{X_c^c}{q_c} \cdot \frac{X_d^c}{q_c} \cdot (\alpha \cdot p).$$

$$\mathbb{P}\left(\begin{array}{ccc} \mathrm{D} & & \\ | & & | \\ \mathrm{D} & \longleftarrow & \mathrm{C} \end{array}\right) = \mathbb{P}\left(\begin{array}{ccc} \mathrm{D} & & \mathrm{C} \\ | & & | \\ \mathrm{D} & \longleftarrow & \mathrm{C} \end{array}\right) + \mathbb{P}\left(\begin{array}{ccc} \mathrm{D} & & \mathrm{D} \\ | & & | \\ \mathrm{D} & \longleftarrow & \mathrm{C} \end{array}\right)$$

$$= \frac{1}{2} \cdot \phi_{c\to d} \cdot p_d \cdot p_{c|d} \cdot \frac{X_d^d}{p_d} \cdot \left(\frac{X_c^c}{p_c} + \frac{X_c^d}{p_c} \cdot (1 - p + \alpha \cdot p)\right).$$

$$\mathbb{P}\left(\begin{array}{ccc} \mathrm{D} & & \mathrm{C} \\ | & & | \\ \mathrm{D} & \leftarrow & \mathrm{D[C]} \end{array}\right) = \frac{1}{2} \cdot \phi_{c \to d} \cdot p_d \cdot p_{d|d} \cdot \frac{X_d^d}{p_d} \cdot \frac{X_d^c}{p_d} \cdot ((1-\alpha) \cdot p).$$
